# Supplementary material for: Phase II trial of cisplatin, gemcitabine and pembrolizumab for platinum-resistant ovarian cancer
Source: PLoS One. 2021 Jun 3;16(6):e0252665. doi: 10.1371/journal.pone.0252665 (PMC8174738; doi:10.1371/journal.pone.0252665)
Supplement: S2 Protocol — (PDF) [file pone.0252665.s003.pdf]

A phase II study of pembrolizumab with cisplatin and gemcitabine treatment in patients with recurrent platinum-resistant ovarian cancer

Principal Investigator: Dr. Christine Walsh, MD
Cedars-Sinai Medical Center
8700 Beverly Blvd.
Los Angeles, CA 90048
310-423-5456; Fax: 310-423-0155
Christine.Walsh@cshs.org

Consenting Co-Investigator(s):

Name	Department/Division
Ilana Cass, MD	Gynecologic Oncology
Andrew Li, MD	Gynecologic Oncology
BJ Rimel, MD	Gynecologic Oncology

Biostatistician: Andre Rogatko, PhD
Director, Biostatistics and Bioinformatics
Cedars Sinai Medical Center
(310) 423-3316
Andre.Rogatko@cshs.org

Study Drug: Pembrolizumab, cisplatin and gemcitabine

IND Number: N/A

Funding Source: Merck

Version: Version 11: 02 Jan 2019

Signature Page

Protocol Version 11: 02Jan2019

The signature below constitutes the approval of this protocol and the attachments, and provides the necessary assurances that this trial will be conducted according to all stipulations of the protocol, including all statements regarding confidentiality, and according to local legal and regulatory requirements and applicable U.S. federal regulations and ICH guidelines.

Principal Investigator (PI) Name: _____________________________

PI Signature: _____________________________

Date: ____________________

TABLE OF CONTENTS

LIST OF ABBREVIATIONS 6
1.0 TRIAL SUMMARY 8
2.0 TRIAL DESIGN 9
2.1 Trial Design 9
2.2 Trial Diagram 9
3.0 OBJECTIVE(S) & HYPOTHESIS(ES) 9
3.1 Primary Objective(s) & Hypothesis(es) 9
3.2 Secondary Objective(s) & Hypothesis(es) 10
3.3 Exploratory Objective 10
4.0 BACKGROUND & RATIONALE 10
4.1 Background 10
4.2 Rationale 11
5.0 METHODOLOGY 15
5.1 Entry Criteria 15
5.2 Trial Treatments 18
5.3 Randomization or Treatment Allocation 24
5.4 Stratification 24
5.5 Concomitant Medications/Vaccinations (allowed & prohibited) 24
5.6 Rescue Medications & Supportive Care 25
5.7 Diet/Activity/Other Considerations 30
5.8 Subject Withdrawal/Discontinuation Criteria 31
5.9 Subject Replacement Strategy 32

5.10 Clinical Criteria for Early Trial Termination 32
6.0 TRIAL FLOW CHART 33
6.1 Study Flow Chart 33
7.0 TRIAL PROCEDURES 35
7.1 Trial Procedures 35
7.2 Assessing and Recording Adverse Events 42
7.3 Reporting Requirements for Adverse Events 46
8.0 STATISTICAL ANALYSIS PLAN 51
8.1 Trial Design 51
8.2 Early Stopping for Futility 51
8.3 Design Operating Characteristics 52
8.4 Early Stopping for Safety 52
8.5 Statistical Analyses 54
9.0 LABELING, PACKAGING, STORAGE AND RETURN OF COMMERCIAL SUPPLIES 54
9.1 Investigational Product 54
9.2 Packaging and Labeling Information 55
9.3 Commercial Supplies Disclosure 55
9.4 Storage and Handling Requirements 55
9.5 Returns and Reconciliation 55
10.0 STUDY MANAGEMENT 55
10.1 Conflict of Interest 55
10.2 Institutional Review Board and Consent 55
10.3 Registration Procedures 56

11.0 Data and Safety monitoring 57
11.1 Data Monitoring and Quality Assurance 57
11.2 Safety Monitoring 57
11.3 Adherence to the Protocol 58
11.4 Amendments to the Protocol 58
11.5 Obligations of Investigators 59
11.6 Record Retention 59
12.0 REFERENCES 60
APPENDIX A: ECOG Performance Status 63
APPENDIX B: CTCAE 64
APPENDIX C: RECIST 1.1 65
APPENDIX D: Immune-Related Respone Criteria 68
APPENDIX E: FACIT-TS-G Questionnaire 69
APPENDIX F: FACT-G Questionnaire 70
APPENDIX G: Summary of Protocol Changes 72

LIST OF ABBREVIATIONS

AE	Adverse Event
ALT	Alanine Aminotransferase
ANC	Absolute neutrophil count
ASCO	American Society of Clinical Oncology
AST	Aspartate Aminotransferase
BUN	Blood Urea Nitrogen
CA125	Cancer Antigen 125
CBC	Complete Blood Count
CD4	Cluster of Differentiation 4
CD8	Cluster of Differentiation 8
CNS	Central Nervous System
CR	Complete Response
CRF	Case Report Form
CrCl	Creatinine Clearance
CRO	Contract Research Organization
CSA	Controlled Substances Act
CSF	Colony-stimulating factor
CT	Computed Tomography
CTC	Common Toxicity Criteria
CTCAE	Common Terminology Criteria for Adverse Events
CTLA-4	Cytotoxic T-lymphocyte-associated protein 4
DKA	Diabetic Ketoacidosis
DLT	Dose Limiting Toxicity
DSMB	Data and Safety Monitoring Board
ECI	Event of Clinical Interest
ECOG	Eastern Cooperative Oncology Group
ERC	Ethical Review Committee
EU	European Union
FACIT-TS-G	Functional Assessment of Chronic Illness Therapy
FACT-G	Functional Assessment of Cancer Therapy
FDA	US Food and Drug Administration
FOXP3	Forkhead Box P3
G-CSF	Granulocyte colony-stimulating factor
GFR	Glomerular Filtration Rate
HIV	Human immunodeficiency virus
Ig	Immunoglobulin
IMM	Independent Medical Monitor
IND	Investigational New Drug
INR	International Normalized Ratio
IRB	Institutional Review Board

irRC	Immune-related response criteria
ITIM	Immunoreceptor Tyrosine-based Inhibition Motif
ITSM	Immunoreceptor Tyrosine-based Switch Motif
IV (or iv)	Intravenously
Ki67	Protein marker for cellular proliferation
LDH	Lactate Dehydrogenase
mAB	Monoclonal antibody
MTD	Maximum Tolerated Dose
NCI	National Cancer Institute
ORR	Overall Response Rate
OS	Overall Survival
OTC	Over the Counter
PD	Progressive Disease
PD-1	Programmed cell death 1
PD-L1	Programmed death-ligand 1
PD-L2	Programmed death-ligand 2
PFS	Progression Free Survival
PK	Pharmacokinetics
PMDA	Pharmaceutical and Medical Devices agency
PO	Per Os (oral)
PR	Partial Response
PT	Prothrombin Time
PTT	Partial Thromboplastin Time
Q	Every
QMC	Quality Management Core
RECIST	Response Evaluation Criteria in Solid Tumors
SAE	Serious Adverse Event
SD	Stable Disease
T1DM	Type 1 Diabetes Mellitus
TB	Tuberculosis
TIL	Tumor Infiltrating Lymphocyte
TSH	Thyroid Stimulating Hormone
ULN	Upper Limit of Normal
WBC	White Blood Cells

1.0 TRIAL SUMMARY

Title	A phase II study of pembrolizumab with cisplatin and gemcitabine treatment in patients with recurrent platinum-resistant ovarian cancer.
Short Title	Pembrolizumab with cisplatin and gemcitabine in platinum-resistant ovarian cancer
Protocol Number	IIT2015-13-Walsh-PemCiGem
Phase	2
Methodology	Single-arm, open-label, phase II trial
Study Duration	24 months (2 years)
Study Center(s)	Single-center
Objectives	To evaluate the efficacy and safety of anti-PD-1 antibody MK-3475 (pembrolizumab) in combination with standard of care gemcitabine and cisplatin chemotherapy in women with recurrent platinum-resistant ovarian cancer
Number of Subjects	25
Diagnosis and Main Inclusion Criteria	Recurrent platinum-resistant ovarian cancer (encompasses ovarian, peritoneal and fallopian tube cancer)
Study Product(s), Dose, Route, Regimen	Intravenous Cisplatin + Gemcitabine + Pembrolizumab
Estimated enrollment period	2/1/2016 – 2/1/2018
Duration of administration	2 cycles of gemcitabine and cisplatin chemotherapy followed by 4 cycles of gemcitabine and cisplatin combined with pembrolizumab in 21-day treatment cycles followed by single-agent pembrolizumab maintenance therapy for up to 2 years of treatment (6 cycles combination treatment + 28 cycles maintenance).
Duration of Participation	Duration of participation will vary. Treatment will continue until disease progression or until the subject meets withdrawal criteria.
Statistical Methodology	Twenty-five patients would be needed to test the null hypothesis: $p \leq 0.111$ against the alternative hypothesis: $p \geq 0.300$ at the 5.0% level of significance and with 80% power.

2.0 TRIAL DESIGN

2.1 Trial Design

This is a single-arm, open-label, phase II trial to evaluate the efficacy and safety of anti-PD-1 antibody MK-3475 (pembrolizumab) in combination with standard of care gemcitabine and cisplatin chemotherapy in women with recurrent platinum-resistant ovarian cancer (encompasses ovarian, peritoneal and fallopian tube cancer). Subjects will receive 2 cycles of gemcitabine and cisplatin chemotherapy followed by 4 cycles of gemcitabine and cisplatin combined with pembrolizumab in 21-day treatment cycles. Subjects will continue to receive single-agent pembrolizumab every 21 days as maintenance therapy for up to an additional 28 cycles until progression or the subject meets withdrawal criteria. Altogether, the maintenance cycles in addition to the 6 combination cycles will total to approximately two years of study treatment. Tumor imaging with CT scan will occur at baseline and every 6 weeks (after each second cycle) during chemotherapy treatment and every 9 weeks during the maintenance phase (or earlier than every 6-9 weeks as clinically indicated per PI discretion). The primary endpoint is efficacy as defined overall response rate by Response Evaluation Criteria in Solid Tumors (RECIST v.1.1). Secondary endpoints for efficacy include progression free survival at 6 and 12 months, time to progression, duration of response and overall survival. Safety and tolerability of the regimen will be determined by assessing the frequency and intensity of adverse events as defined by the Common Terminology Criteria for Adverse Events (CTCAE v.4). Quality of life will be measured using the FACIT-TS-G and FACT-G (See APPENDIX).

2.2 Trial Diagram

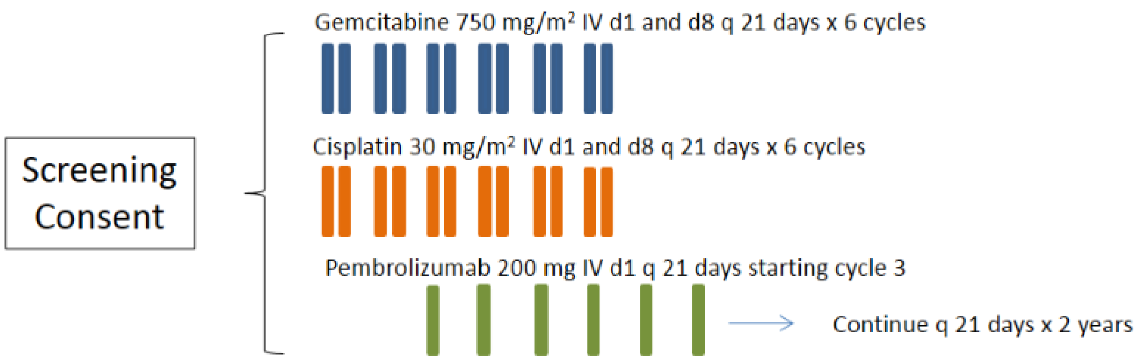

3.0 OBJECTIVE(S) & HYPOTHESIS(ES)

3.1 Primary Objective(s) & Hypothesis(es)

(1) **Objective:** To estimate the anti-tumor activity and durability of response (definitions in section 4.2.3.1) to pembrolizumab with cisplatin and gemcitabine treatment in patients with recurrent, platinum-resistant ovarian cancer as determined by Response Evaluation Criteria in Solid Tumors (RECIST).

Hypothesis: The combination of pembrolizumab with standard of care cisplatin and gemcitabine treatment in patients with recurrent, platinum-resistant ovarian cancer will result in durable objective responses.

3.2 Secondary Objective(s) & Hypothesis(es)

(1) **Objective**: To determine the safety and tolerability of pembrolizumab with cisplatin and gemcitabine treatment in patients with recurrent, platinum-resistant ovarian cancer as assessed by Common Terminology Criteria for Adverse Events (CTCAE).

Hypothesis: The combination of pembrolizumab with standard of care cisplatin and gemcitabine treatment in patients with recurrent, platinum-resistant ovarian cancer will be well tolerated.

3.3 Exploratory Objective

(1) **Objective:** To explore whether baseline tumor characteristics predict response to the combination of pembrolizumab with standard of care cisplatin and gemcitabine treatment in patients with recurrent, platinum-resistant ovarian cancer.

4.0 BACKGROUND & RATIONALE

4.1 Background

Refer to the Investigator's Brochure (IB)/approved labeling for detailed background information on MK-3475.

4.1.1 Pharmaceutical and Therapeutic Background

The importance of intact immune surveillance in controlling outgrowth of neoplastic transformation has been known for decades. Accumulating evidence shows a correlation between tumor-infiltrating lymphocytes (TILs) in cancer tissue and favorable prognosis in various malignancies [1-3]. In particular, the presence of CD8+ T-cells and the ratio of CD8+ effector T-cells / FoxP3+ regulatory T-cells seems to correlate with improved prognosis and long-term survival in many solid tumors [4].

The PD-1 receptor-ligand interaction is a major pathway hijacked by tumors to suppress immune control. The normal function of PD-1, expressed on the cell surface of activated T-cells under healthy conditions, is to down-modulate unwanted or excessive immune responses, including autoimmune reactions. PD-1 (encoded by the gene Pdcd1) is an Ig superfamily member related to CD28 and CTLA-4 which has been shown to negatively regulate antigen receptor signaling upon engagement of its ligands (PD-L1 and/or PD-L2) [5]. The structure of murine PD-1 has been resolved. PD-1 and family members are type I transmembrane glycoproteins containing an Ig Variable-type (V-type) domain responsible for ligand binding and a cytoplasmic tail which is responsible for the binding of signaling molecules. The cytoplasmic tail of PD-1 contains 2 tyrosine-based signaling motifs, an immunoreceptor tyrosine-based inhibition motif (ITIM) and an immunoreceptor tyrosine-based switch motif (ITSM). Following T-cell stimulation, PD-1 recruits the tyrosine phosphatases SHP-1 and SHP-2 to the ITSM motif within its cytoplasmic tail, leading to the dephosphorylation of effector molecules such as CD3ζ, PKCθ and ZAP70 which are involved in the CD3 T-cell signaling cascade. The mechanism by which PD-1 down modulates T-cell responses is similar to, but distinct from that of CTLA-4 as both molecules regulate an overlapping set of signaling proteins. PD-1 was shown to be expressed on activated lymphocytes including peripheral CD4+ and CD8+ T-cells, B-cells, T regs and Natural Killer cells. Expression has also been shown during thymic development on CD4-CD8- (double negative) T-cells as well as subsets of macrophages and dendritic cells. The ligands for PD-1 (PD-L1 and PD-L2) are constitutively expressed or can be induced in a variety of cell types, including non-hematopoietic tissues as well as in various tumors. Both ligands are type I transmembrane receptors containing both IgV- and IgC-like domains in the extracellular region and contain short cytoplasmic regions with no known

signaling motifs. Binding of either PD-1 ligand to PD-1 inhibits T-cell activation triggered through the T-cell receptor. PD-L1 is expressed at low levels on various non-hematopoietic tissues, most notably on vascular endothelium, whereas PD-L2 protein is only detectably expressed on antigen-presenting cells found in lymphoid tissue or chronic inflammatory environments. PD-L2 is thought to control immune T-cell activation in lymphoid organs, whereas PD-L1 serves to dampen unwarranted T-cell function in peripheral tissues. Although healthy organs express little (if any) PD-L1, a variety of cancers were demonstrated to express abundant levels of this T-cell inhibitor. PD-1 has been suggested to regulate tumor-specific T-cell expansion in subjects with melanoma. This suggests that the PD-1/PD-L1 pathway plays a critical role in tumor immune evasion and should be considered as an attractive target for therapeutic intervention.

Pembrolizumab is a potent and highly selective humanized monoclonal antibody (mAb) of the IgG4/kappa isotype designed to directly block the interaction between PD-1 and its ligands, PD-L1 and PD-L2. In September, 2014, Keytruda™ (pembrolizumab) was approved in the United States for the treatment of patients with unresectable or metastatic melanoma and disease progression following ipilumumab and, if BRAF V600 mutation positive, a BRAF inhibitor. Keytruda™ was also granted accelerated approval in October 2015 by the FDA to treat patients with advanced non-small cell lung cancer whose disease has progressed after other treatments and with tumors that express PD-L1.

4.1.2 Preclinical and Clinical Trial Data

Refer to the Investigator’s Brochure for Preclinical and Clinical data.

4.2 Rationale

4.2.1 Rationale for the Trial and Selected Subject Population

Ovarian cancer is the most lethal of the gynecologic malignancies. While 75% of patients with advanced epithelial ovarian cancer will achieve a clinical remission following surgical cytoreduction and platinum and taxane based chemotherapy, the vast majority will relapse and develop chemoresistant disease [6]. Despite aggressive surgical and chemotherapeutic treatment, fewer than 50% of patients with stage III or IV disease will survive longer than 5 years. There is a critical need for more effective therapies for this fatal malignancy.

In solid tumors including melanoma, lung, kidney, prostate and other cancers, modulation of the immune system is showing promise as an effective treatment strategy [7]. This is an active area of research in many tumor types and the optimal immunotherapy approach for ovarian cancer patients has yet to be defined.

Therapeutic blockade of immune checkpoints releases the silencing of cytotoxic T-cell responses and allows for effective anti-tumor immune responses. In an earlier study, antibody treatment against PD-L1 (BMS-936559) caused tumor regression and prolonged disease stabilization in heavily pretreated patients with a variety of solid tumors and also showed a milder toxicity profile compared to anti-CTLA-4 antibody treatment [8]. However, in this trial, only 1 of 17 patients with advanced ovarian cancer demonstrated an objective response. In 2015, Hamanishi and colleagues reported data on the efficacy and safety of an anti-PD-1 antibody (BMS-936558, nivolumab) in patients with platinum-resistant ovarian cancer [9, 10]. Used as a single agent, the anti-PD-1 treatment was found to be well tolerated and associated with a 15% best overall response rate and 45% disease control rate. The median overall survival time was 20 months at study termination. Taken together, these data suggest that immune checkpoint blockade may have efficacy in the treatment of ovarian cancer.

Platinum-resistance correlates highly with resistance to other cytotoxic therapies. When ovarian cancer relapses within 6 months of last chemotherapy, response rates to later-line agents range from 10-20%. One of the more effective treatment options in this setting has been the combination use of cisplatin and gemcitabine chemotherapy. Laboratory studies demonstrate synergy between the compounds

and the ability of gemcitabine to reverse platinum resistance [11]. There have been three clinical trials testing the combination of cisplatin and gemcitabine in platinum-resistant ovarian cancer populations. The results are summarized below.

1. Rose, et al. Gynecol Oncol 2003 [12]. In this single institution study, 36 platinum-resistant ovarian cancer patients (multiple prior chemotherapy regimens eligible) were treated with gemcitabine 750 mg/m2 + cisplatin 30 mg/m2 on days 1 and 8 of a 21-day cycle. There were 4 (11%) complete responses, 11 (31%) partial responses, 9 (26%) stable disease and 11 (31) progressive disease. Among the responders, the median duration of response was 11 months. Progression-free interval was 6 months and median overall survival was 12 months. Responses were seen, even in the setting of prior gemcitabine exposure. Four of six patients who failed prior gemcitabine treatment were responders to the regimen.

2. Nagourney, et al. Gynecol Oncol 2003 [13]. In this single institution study, 27 patients with platinum-resistant ovarian cancer (multiple prior chemotherapy regimens eligible) were treated with cisplatin 30 m/gm2 and gemcitabine 600-750 mg/m2 on days 1 and 8 of a 21-day cycle. There were 7 (26%) complete responses, 12 (44%) partial responses, 7 (26%) stable disease and 1 (4%) disease progression. The median time to progression for objective responders was 8 months.

3. Brewer, et al. Gynecol Oncol 2006 [14]. In this multi-center cooperative group, GOG trial (GOG 126-L), 59 patients with platinum-resistant ovarian cancer (eligibility limited to one prior platinum-based regimen) were treated with cisplatin 30 mg/m2 followed by gemcitabine 750 mg/m2. The dose of gemcitabine was reduced to 600 mg/m2 due to hematologic toxicity. There were 4 (7%) complete responses, 5 (9%) partial responses, 31 (54%) stable disease and 12 (21%) progressive disease. The median time to progression was 5.4 months. The median overall survival was 15 months.

Taken together, these three studies suggest activity for the combination of cisplatin and gemcitabine in the platinum-resistant and gemcitabine-resistant ovarian cancer population. The sequencing of the two drugs appears to impact the toxicity profile. Gemcitabine given prior to cisplatin appears to result in less hematologic toxicity and better tolerability. Despite promising response rates, the durability of response was limited, ranging from 5.4 to 8 months in the three trials. The purpose of this study is to determine whether the addition of an anti-PD-1 monoclonal antibody to cisplatin and gemcitabine treatment will improve upon the responses and durability of remissions in patients with platinum-resistant ovarian cancer.

The body of literature addressing the combination of immunotherapy with chemotherapy is growing. Because chemotherapy-induced lymphodepletion is predicted to limit the ability to mount an immune response, the combination of chemotherapy with immunotherapy has been thought to be antagonistic. However, there is data to suggest that chemotherapy and immunotherapy could work synergistically when sequenced properly. Established tumors employ a number of powerful immunosuppressive mechanisms to escape immune surveillance [5]. Chemotherapy reverses suppression by causing loss of suppressive T-regulatory cells and triggers a “re-booting” of the immune system that causes T-cells to become more susceptible to antigen-recognition [15-19]. Removal of tumor burden relieves the immune system from persistent antigen presentation which subsequently removes tolerance and allows T cells to regain anti-tumor function [20].

Cisplatin and gemcitabine also have specific impacts on the immune system. Cisplatin decreases T-regulatory and myeloid derived suppressor cells (MDSC) and sensitizes tumor cells to T cell-mediated lysis [21, 22]. Gemcitabine downregulates MDSC and has direct immunostimulatory effects [23, 24].

Clinical trials combining immune checkpoint inhibitors with chemotherapy have recently been reported and the combination demonstrates anti-tumor activity with acceptable toxicity. A phase I study combining nivolumab (anti-PD-1) with platinum-based doublet chemotherapy (cisplatin/gemcitabine, cisplatin/pemetrexed, carboplatin/paclitaxel) in non-small cell lung cancer demonstrated antitumor activity, no dose-limiting toxicities during the first 6 weeks of treatment, an acceptable toxicity profile and encouraging 1 year overall survival (ASCO 2014, NCT01454102) [25]. Nivolumab and

pembrolizumab have similar toxicity profiles [26]. As the safety of anti-PD-1 inhibition with cisplatin and gemcitabine has been demonstrated in this prior trial (no dose limiting toxicities), we plan to study the combination of pembrolizumab with the cisplatin/gemcitabine platinum doublet in this trial with a phase II design with early stopping rules for safety.

Additional research suggests that the phasing of immunotherapy with chemotherapy has an impact on efficacy of the combination. A phase II study combining the anti-CTLA4 antibody ipilimumab with paclitaxel and carboplatin in first-line treatment of advanced non-small cell lung cancer showed no improvement in progression-free survival (PFS) when ipilimumab was administered concurrently with chemotherapy compared to chemotherapy alone. However, when ipilimumab was added after two rounds of chemotherapy in a phased regimen that allowed for antigen release to occur prior to immunotherapy exposure, there was an improvement in PFS [27].

Based on this prior experience, we will evaluate the impact of adding the anti-PD-1 antibody MK-3475 (pembrolizumab) to standard of care cisplatin and gemcitabine chemotherapy in the treatment of patients with platinum-resistant ovarian cancer. We will administer two cycles of chemotherapy to allow for tumor cytoreduction, tumor antigen release, decrease in suppressive T-regulatory and myeloid derived suppressor cells and remodeling of the tumor microenvironment. Pembrolizumab will be added to cycles 3-6 of chemotherapy and then will be continued as single-agent maintenance therapy every 3 weeks for up to one year until disease progression or subject meets withdrawal criteria.

4.2.2 Rationale for Dose Selection/Regimen/Modification

An open-label Phase I trial (Protocol 001) has been conducted by Merck to evaluate the safety and clinical activity of single agent MK-3475. The dose escalation portion of this trial evaluated three dose levels, 1 mg/kg, 3 mg/kg, and 10 mg/kg, administered every 2 weeks (Q2W) in subjects with advanced solid tumors. All three dose levels were well tolerated and no dose-limiting toxicities were observed. This first in human study of MK-3475 showed evidence of target engagement and objective evidence of tumor size reduction at all dose levels (1 mg/kg, 3 mg/kg and 10 mg/kg Q2W). No MTD has been identified to date. 10.0 mg/kg Q2W, the highest dose tested in PN001, will be the dose and schedule utilized in Cohorts A, B, C and D of this protocol to test for initial tumor activity. Recent data from other clinical studies within the MK-3475 program has shown that a lower dose of MK-3475 and a less frequent schedule may be sufficient for target engagement and clinical activity.

PK data analysis of MK-3475 administered Q2W and Q3W showed slow systemic clearance, limited volume of distribution, and a long half-life (refer to IB). Pharmacodynamic data (IL-2 release assay) suggested that peripheral target engagement is durable (>21 days). This early PK and pharmacodynamic data provides scientific rationale for testing a Q2W and Q3W dosing schedule.

A population pharmacokinetic analysis has been performed by Merck using serum concentration time data from 476 patients. Within the resulting population PK model, clearance and volume parameters of MK-3475 were found to be dependent on body weight. The relationship between clearance and body weight, with an allometric exponent of 0.59, is within the range observed for other antibodies and would support both body weight normalized dosing or a fixed dose across all body weights. MK-3475 has been found to have a wide therapeutic range based on the melanoma indication. The differences in exposure for a 200 mg fixed dose regimen relative to a 2 mg/kg Q3W body weight based regimen are anticipated to remain well within the established exposure margins of 0.5 – 5.0 for MK-3475 in the melanoma indication. The exposure margins are based on the notion of similar efficacy and safety in melanoma at 10 mg/kg Q3W vs. the proposed dose regimen of 2 mg/kg Q3W (i.e. 5-fold higher dose and exposure). The population PK evaluation revealed that there was no significant impact of tumor burden on exposure. In addition, exposure was similar between the NSCLC and melanoma indications. Therefore, there are no anticipated changes in exposure between different indication settings.

The rationale for further exploration of 2 mg/kg and comparable doses of pembrolizumab in solid tumors is based on: 1) similar efficacy and safety of pembrolizumab when dosed at either 2 mg/kg or 10 mg/kg Q3W in melanoma patients, 2) the flat exposure-response relationships of pembrolizumab for both efficacy and safety in the dose ranges of 2 mg/kg Q3W to 10 mg/kg Q3W, 3) the lack of effect of tumor burden or indication on distribution behavior of pembrolizumab (as assessed by the population PK model) and 4) the assumption that the dynamics of pembrolizumab target engagement will not vary meaningfully with tumor type.

The choice of the 200 mg Q3W as an appropriate dose for the switch to fixed dosing is based on simulations performed using the population PK model of pembrolizumab showing that the fixed dose of 200 mg every 3 weeks will provide exposures that 1) are optimally consistent with those obtained with the 2 mg/kg dose every 3 weeks, 2) will maintain individual patient exposures in the exposure range established in melanoma as associated with maximal efficacy response and 3) will maintain individual patients exposure in the exposure range established in melanoma that are well tolerated and safe.

4.2.3 Rationale for Endpoints

We will assess the efficacy and safety of pembrolizumab with cisplatin and gemcitabine chemotherapy in patients with platinum-resistant ovarian cancer. We will evaluate overall response rate as the primary efficacy endpoint and progression-free survival at 6 and 12 months, time to progression, duration of response and overall survival as secondary efficacy endpoints. Efficacy will be determined by imaging at baseline and every 2 cycles during chemotherapy and every 3 cycles thereafter (or earlier as clinically indicated per PI discretion) until disease progression or treatment discontinuation. Response will be determined by RECIST 1.1 andiRECIST. To determine safety, we will report the frequency and intensity of adverse events by Common Terminology for Adverse Events (CTCAE v.4 criteria).

4.2.3.1 Efficacy and Safety Endpoints

1. Overall response rate (Proportion of patients who achieve a complete or partial response per RECIST 1.1 criteria).

2. Overall response rate by iRECIST[28].

3. Progression-free survival at 6 months (Proportion of patients who have not progressed at 6 months with progression-free survival calculated from the start of treatment to the date of progression or death from any cause).

4. Progression-free survival at 12 months (Proportion of patients who have not progressed at 12 months with progression-free survival calculated from the start of treatment to the date of progression or death from any cause).

5. Time to progression (calculated in months from the start of treatment to disease progression as defined by RECIST 1.1).

6. Duration of response (calculated in months as time from documentation of tumor response to disease progression).

7. Overall survival (calculated in months from the start of treatment to the date of death from any cause).

8. Frequency and intensity of adverse events (CTCAE v.4) as measured at each visit, during safety follow up (30 days after discontinuation of treatment) and during follow up (every nine weeks after discontinuation)

4.2.3.2 Biomarker Research

Subjects will be given the option to participate in optional biomarker research.

Subjects will be given the option to consent for optional CT-guided biopsy prior to treatment. Formalin-fixed, paraffin-embedded slides will be obtained from the primary cytoreductive surgery if available as disclosed in the main informed consent form.

Tissue specimens will be assessed for PD-L1 expression by immunohistochemistry. Other biomarkers may also be tested including, but not limited to PD-1, CD8, CD4, FoxP3 and Ki67. Immunohistochemistry expression will be correlated to treatment response in an exploratory manner.

Subjects will be given the opportunity to consent for optional blood collection. If the patient provides consent, blood will be collected at baseline and every 2 cycles for storage for future correlative studies.

4.2.3.3 Quality of Life

Quality of life (QOL) will be measured by the FACIT-TS-G and FACT-G questionnaires [29]. The FACT-G questionnaire will be administered at baseline. The FACT-G and FACIT-TS-G questionnaires will be administered between cycle 2 - 3 (reflecting QOL after 2 cycles of chemotherapy), between cycle 4 - 5 (reflecting QOL after 2 cycles of chemotherapy + pembrolizumab) and at 6 (between cycle 8 and 9), 12 months (after cycle 17) and 24 months (after cycle 34) during maintenance therapy.

5.0 METHODOLOGY

5.1 Entry Criteria

5.1.1 Subject Inclusion Criteria

5.1.1.1 **In order to be eligible for participation in this trial, the subject must:** Be willing and able to provide written informed consent/ for the trial.

5.1.1.2 Be ≥ 18 years of age on day of signing informed consent.

5.1.1.3 Have diagnosis of recurrent epithelial ovarian, peritoneal or fallopian tube carcinoma that has progressed within 6 months of prior cytotoxic platinum-based chemotherapy. Histologic confirmation of the primary tumor by review of the pathology report is required. Patients must have had at least one prior platinum-based chemotherapeutic regimen. Initial treatment may have been administered as an intraperitoneal, intravenous or dose-dense regimen. Progression on a non-platinum containing regimen is eligible if the patient is considered platinum-resistant to the last platinum-containing regimen. Patients who have received prior cisplatin and gemcitabine treatment are eligible to participate.

5.1.1.4 Have measurable disease based on RECIST 1.1 (See Appendix C)

5.1.1.5 Have a performance status of 0 or 1 on the ECOG Performance Scale.

5.1.1.6 Demonstrate adequate organ function as defined in Table 1, all screening labs should be performed within 28 days of treatment initiation.

5.1.1.7 Female subject of childbearing potential should have a negative urine or serum pregnancy test within 72 hours prior to receiving the first dose of study medication. If the urine test is positive or cannot be confirmed as negative, a serum pregnancy test will be required.

Patients who have had prior hysterectomy and/or bilateral oophorectomy are not required to have a pregnancy test.

5.1.1.8 Female subjects of childbearing potential should be willing to use 2 methods of birth control or be surgically sterile, or abstain from heterosexual activity for the course of the study through 120 days after the last dose of study medication (Reference Section 5.7.2). Subjects of childbearing potential are those who have not been surgically sterilized or have not been free from menses for > 1 year.

Table 1 Adequate Organ Function Laboratory Values

System	Laboratory Value
**Hematological**	
Absolute neutrophil count (ANC)	≥1,500 /mcL
Platelets	≥100,000 / mcL
Hemoglobin	≥9 g/dL or ≥5.6 mmol/L without transfusion or EPO dependency (within 7 days of assessment)
**Renal**	
Serum creatinine **OR**<br>Measured or calculated[a] creatinine clearance<br>(GFR can also be used in place of creatinine or CrCl)	≤1.5 X upper limit of normal (ULN) **OR**<br><br>≥60 mL/min for subject with creatinine levels > 1.5 X institutional ULN
**Hepatic**	
Serum total bilirubin	≤ 1.5 X ULN **OR**
	Direct bilirubin ≤ ULN for subjects with total bilirubin levels > 1.5 ULN
AST (SGOT) and ALT (SGPT)	≤ 2.5 X ULN **OR**<br>**≤** 5 X ULN for subjects with liver metastases
Albumin	≥2.5 mg/dL
**Coagulation**	
International Normalized Ratio (INR) or Prothrombin Time (PT)	≤1.5 X ULN unless subject is receiving anticoagulant therapy as long as PT or PTT is within therapeutic range of intended use of anticoagulants
Activated Partial Thromboplastin Time (aPTT)	≤1.5 X ULN unless subject is receiving anticoagulant therapy as long as PT or PTT is within therapeutic range of intended use of anticoagulants
[a]Creatinine clearance should be calculated per institutional standard.	

5.1.2 Subject Exclusion Criteria

The subject must be excluded from participating in the trial if the subject:

5.1.2.1 Is currently participating and receiving study therapy or has participated in a study of an investigational agent and received study therapy or used an investigational device within 4 weeks of the first dose of treatment.

5.1.2.2 Has diagnosis of immunodeficiency or is receiving systemic steroid therapy or any other form of immunosuppressive therapy within 7 days prior to the first dose of trial treatment.

5.1.2.3 Has a known history of active TB (Bacillus Tuberculosis)

5.1.2.4 Hypersensitivity to pembrolizumab or any of its excipients. Has had a prior anti-cancer monoclonal antibody (mAb) within 4 weeks prior to study Day 1 or who has not recovered (i.e., ≤ Grade 1 or at baseline) from adverse events due to agents administered more than 4 weeks earlier.

5.1.2.5 Has had prior chemotherapy, targeted small molecule therapy, or radiation therapy within 2 weeks prior to study Day 1 or who has not recovered (i.e., ≤ Grade 1 or at baseline) from adverse events due to a previously administered agent.

- Note: Subjects with ≤ Grade 2 neuropathy are an exception to this criterion and may qualify for the study.
- Note: If subject received major surgery including (curative or palliative surgery), they must have recovered adequately from the toxicity and/or complications from the intervention prior to starting therapy.
- Note: Patients who have hypertension as an adverse event related to prior angiogenesis targeted therapy may be allowed if ≤ Grade 2 and considered by investigator to be well-controlled on anti-hypertensive agents.

5.1.2.6 Has a known additional malignancy that is progressing or requires active treatment. Exceptions include basal cell carcinoma of the skin or squamous cell carcinoma of the skin that has undergone potentially curative therapy or in situ cervical cancer.

5.1.2.7 Has known active central nervous system (CNS) metastases and/or carcinomatous meningitis. Subjects with previously treated brain metastases may participate provided they are stable (without evidence of progression by imaging for at least four weeks prior to the first dose of trial treatment and any neurologic symptoms have returned to baseline), have no evidence of new or enlarging brain metastases, and are not using steroids for at least 7 days prior to trial treatment. This exception does not include carcinomatous meningitis which is excluded regardless of clinical stability.

5.1.2.8 Has active autoimmune disease that has required systemic treatment in the past 2 years (i.e. with use of disease modifying agents, corticosteroids or immunosuppressive drugs). Replacement therapy (e.g., thyroxine, insulin, or physiologic corticosteroid replacement therapy for adrenal or pituitary insufficiency, etc.) is not considered a form of systemic treatment.

5.1.2.9 Has a history of (non-infectious) pneumonitis that required steroids or current pneumonitis.

5.1.2.10 Has an active infection requiring systemic therapy.

5.1.2.11 Has a history or current evidence of any condition, therapy, or laboratory abnormality that might confound the results of the trial, interfere with the subject’s participation for the full

duration of the trial, or is not in the best interest of the subject to participate, in the opinion of the treating investigator.

5.1.2.12 Has known psychiatric or substance abuse disorders that would interfere with cooperation with the requirements of the trial.

5.1.2.13 Is pregnant or breastfeeding, or expecting to conceive children within the projected duration of the trial, starting with screening visit through 120 days after the last dose of trial treatment.

5.1.2.14 Has received prior therapy with an anti-PD-1, anti-PD-L1, or anti-PD-L2 agent.

5.1.2.15 Has a known history of Human Immunodeficiency Virus (HIV) (HIV 1/2 antibodies) or positive serum test for HIV as per testing at screening

5.1.2.16 Has known active Hepatitis B (e.g., HBsAg reactive) or Hepatitis C (e.g., HCV RNA [qualitative] is detected) as per test at screening

5.1.2.17 Has received a live vaccine within 30 days of planned start of study therapy.

- *Note: Seasonal influenza vaccines for injection are generally inactivated flu vaccines and are allowed; however intranasal influenza vaccines (e.g., Flu-Mist®) are live attenuated vaccines, and are not allowed.*

5.2 Trial Treatments

The treatment to be used in this trial is outlined below in Table 2

Table 2 Trial Treatment

Drug	Dose/Potency	Dose Frequency	Route of Administration	Regimen/Treatment Period	Use
Gemcitabine	750 mg/m^2	Q 3W x 6 cycles	IV infusion	Day 1 and Day 8 of each 3-week cycle	Standard of care
Cisplatin	30 mg/m^2	Q 3W x 6 cycles	IV infusion	Day 1 and Day 8 of each 3-week cycle after gemcitabine	Standard of care
Pembrolizumab	200 mg	Q3W starting with cycle 3	IV infusion	Day 1 of each 3-week cycle after gemcitabine and cisplatin	Experimental

5.2.1 Dose Selection/Modification

5.2.1.1 Dose Selection

The rationale for selection of doses to be used in this trial is provided in Section 4.0 – Background and Rationale.

Details on preparation and administration of pembrolizumab (MK-3475) are provided in the Pharmacy Manual.

5.2.1.2 Dose Modification

Adverse events (both non-serious and serious) associated with pembrolizumab exposure may represent an immunologic etiology. These adverse events may occur shortly after the first dose or several months after the last dose of treatment. Pembrolizumab must be withheld for drug-related toxicities and severe or life-threatening AEs as per Table 3A below and per Appendix G.

See Appendix G for dose modification and toxicity management guidelines for immune-related AEs associated with Pembrolizumab.

See Section 5.6.1 and Events of Clinical Interest Guidance Document for supportive care guidelines, including use of corticosteroids.

Dosing interruptions are permitted in the case of medical / surgical events or logistical reasons not related to study therapy (e.g., elective surgery, unrelated medical events, patient vacation, and/or holidays). Subjects should be placed back on study therapy within 3 weeks of the scheduled interruption, unless otherwise discussed with the PI. The reason for interruption should be documented in the patient's study record.

A maximum of two dose level modification to chemotherapy is permitted before withdrawing the patient from further chemotherapy, although patient may continue on Pembrolizumab monotherapy, should they not tolerate the combination treatment.

Table 3A: Dose hold and discontinuation for pembrolizumab Guidelines for Drug-Related Adverse Events

Toxicity	Hold Treatment For Grade	Timing for Restarting Treatment	Discontinue Subject
Diarrhea/Colitis	2-3	Toxicity resolves to Grade 0-1.	Toxicity does not resolve within 12 weeks of last dose or inability to reduce corticosteroid to 10 mg or less of prednisone or equivalent per day within 12 weeks.
	4	Permanently discontinue	Permanently discontinue
AST, ALT, or Increased Bilirubin	2	Toxicity resolves to Grade 0-1	Toxicity does not resolve within 12 weeks of last dose.
	3-4	Permanently discontinue (see exception below)[1]	Permanently discontinue
Type 1 diabetes mellitus (if new onset) or Hyperglycemia	T1DM or 3-4	Hold pembrolizumab for new onset Type 1 diabetes mellitus or Grade 3-4 hyperglycemia associated with evidence of beta cell failure.	Resume pembrolizumab when patients are clinically and metabolically stable.
Hypophysitis	2-3	Toxicity resolves to Grade 0-1	Toxicity does not resolve within 12 weeks of last dose or inability to reduce corticosteroid to 10 mg or less of prednisone or equivalent per day within 12 weeks.
	4	Permanently discontinue	Permanently discontinue
Hyperthyroidism	3	Toxicity resolves to Grade 0-1	Toxicity does not resolve within 12 weeks of last dose or inability to reduce corticosteroid to 10 mg or less of prednisone or equivalent per day within 12 weeks.
	4	Permanently discontinue	Permanently discontinue

Toxicity	Hold Treatment For Grade	Timing for Restarting Treatment	Discontinue Subject
Hypothyroidism		Therapy with pembrolizumab can be continued while treatment for the thyroid disorder is instituted	Therapy with pembrolizumab can be continued while treatment for the thyroid disorder is instituted.
Infusion Reaction	3-4	Permanently discontinue	Permanently discontinue
Pneumonitis	2	Toxicity resolves to Grade 0-1	Toxicity does not resolve within 12 weeks of last dose or inability to reduce corticosteroid to 10 mg or less of prednisone or equivalent per day within 12 weeks.
	3-4	Permanently discontinue	Permanently discontinue
Renal Failure or Nephritis	2	Toxicity resolves to Grade 0-1	Toxicity does not resolve within 12 weeks of last dose or inability to reduce corticosteroid to 10 mg or less of prednisone or equivalent per day within 12 weeks.
	3-4	Permanently discontinue	Permanently discontinue
All Other Drug-Related Toxicity[2]	3 or Severe	Toxicity resolves to Grade 0-1	Toxicity does not resolve within 12 weeks of last dose or inability to reduce corticosteroid to 10 mg or less of prednisone or equivalent per day within 12 weeks.
	4	Permanently discontinue	Permanently discontinue

Note: Permanently discontinue for any severe or Grade 3 drug-related AE that recurs or any life-threatening event.

[1] For patients with liver metastasis who begin treatment with Grade 2 AST or ALT, if AST or ALT increases by greater than or equal to 50% relative to baseline and lasts for at least 1 week then patients should be discontinued.

[2] Patients with intolerable or persistent Grade 2 drug-related AE may hold study medication at physician discretion. Permanently discontinue study drug for persistent Grade 2 adverse reactions for which treatment with study drug has been held, that do not recover to Grade 0-1 within 12 weeks of the last dose.

Table 3B: Dose modifications for chemotherapy

	<u>**Gemcitabine**</u>	<u>**Cisplatin**</u>
Starting Dose	750 mg/m^2	30 mg/m^2
1 level reduction	600 mg/m^2	25 mg/m^2
2 level reduction	450 mg/m^2	20 mg/m^2

Treatment decisions will be based upon review of adverse events and laboratory studies drawn prior to each cycle.

Table 3C: Guidelines for chemotherapy dose modifications

Treatment Parameter	Treatment Modification
DAY 1: ANC < 1500 cells/mm^3 and/or platelet count < 100,000/µl	Cycle 1 and 2: Hold cisplatin and gemcitabine chemotherapy. Initiation of cycle 1 or 2 of chemotherapy will be delayed a maximum of three weeks until these threshold values are achieved. Subjects who fail to recover adequate counts within a three-week delay will not receive further chemotherapy in the study.<br><br>Cycle 3 – 6: Hold cisplatin and gemcitabine chemotherapy and administer pembrolizumab only on day 1. Re-assess for chemotherapy administration on day 8.
DAY 8: ANC < 1000 cells/mm^3 and/or platelet count < 75,000/µl	Eliminate day 8 treatment for current cycle.
Febrile neutropenia during prior cycle	Dose reduce gemcitabine 1 level
ANC < 500 cells/mm^3 during prior cycle	Dose reduce gemcitabine 1 level
Platelet count < 50,000/µl during prior cycle	Dose reduce gemcitabine 1 level
Grade 2 or greater peripheral neuropathy (moderate symptoms, limiting instrumental activities of daily living)	Hold cisplatin (administer gemcitabine and/or pembrolizumab if scheduled). Delay subsequent cisplatin therapy for a maximum of 3 weeks until toxicity resolved to grade 1 (asymptomatic, paresthesia). Dose reduce cisplatin 1 level for subsequent cycles.
Grade 2 or greater renal toxicity (serum creatinine greater than 1.8 mg/dL or 1.5X baseline)	Hold cisplatin (administer gemcitabine and/or pembrolizumab if scheduled). Delay subsequent cisplatin therapy for a maximum of 3 weeks until toxicity resolved to grade 1 (serum creatinine ≤ 1.8 mg/dL). Dose reduce cisplatin 1 level for subsequent cycles.
Grade 3 or greater liver toxicity:<br><br>ALT > 225 U/L (5X ULN)<br><br>AST > 175 U/L (5X ULN)<br><br>Alkaline Phosphatase > 625 U/L (5X ULN)<br><br>Total Bilirubin > 3.6 mg/dL (3X ULN)	Dose reduce gemcitabine 1 level. Delay subsequent gemcitabine therapy (but continue to administer cisplatin and/or pembrolizumab if scheduled) for a maximum of 3 weeks until toxicity resolved to grade 1 defined below.<br><br>ALT < 135 U/L (3X ULN)<br><br>AST < 105 U/L (3X ULN)<br><br>Alkaline Phosphatase < 312 U/L (2.5X ULN)<br><br>Total Bilirubin < 1.8 mg/dL (1.5X ULN)

Grade 3 or greater nausea (inadequate oral caloric or fluid intake)<br><br>Grade 3 or greater vomiting (≥ 6 episodes separated by 5 minutes in a 24 hour period)<br><br>Tube feeding, TPN or hospitalization indicated	Optimize antiemetic therapy. If persistent grade 3 toxicity despite optimal antiemetic therapy, dose reduce cisplatin 1 level.
All other grade 3 or 4 toxicity attributable to chemotherapy	Reduce dose, hold dose, or discontinue chemotherapy agent per physician discretion. Continue to administer pembrolizumab if scheduled. May restart chemotherapy treatment when toxicity resolved to grade 0-1 per physician discretion.
Cisplatin and/or gemcitabine treatment delay for more than 3 weeks	Discontinue cisplatin and/or gemcitabine chemotherapy and continue with single agent pembrolizumab

Growth factor support may be administered as per the 2015 American Society of Clinical Oncology Clinical Practice Guideline Update [30] or per investigator discretion.

- Primary prophylaxis with a CSF starting with the first cycle and continuing through subsequent cycles of chemotherapy is recommended in patients who have an approximately 20% or higher risk for febrile neutropenia based on patient-, disease-, and treatment-related factors. In addition to chemotherapy regimen and type of malignancy, consider the following factors when estimating the patient's overall risk of febrile neutropenia: age ≥ 65 years, advanced disease, previous chemotherapy or radiation therapy, preexisting neutropenia or bone marrow involvement with tumor, infection, open wounds or recent surgery, poor nutritional status, poor renal function, liver dysfunction (most notably elevated bilirubin), cardiovascular disease, multiple comorbid conditions.

- Secondary prophylaxis with a CSF is recommended for patients who experienced a neutropenic complication from a prior cycle of chemotherapy (for which primary prophylaxis was not received).

- CSFs should be considered in patients with fever and neutropenia who are at high risk for infection-associated complications or who have prognostic factors predictive or poor clinical outcomes. High-risk features include expected prolonged (> 10 days) and profound (ANC < 100 cells/mm^3) neutropenia, age > 65 years, uncontrolled primary disease, pneumonia, hypotension and multiorgan dysfunction (sepsis syndrome), invasive fungal infection or hospitalization at the time of fever development.

- Pegfilgrastim, filgrastim, tbo-filgrastim, and filgrastim-sndz (and other biosimilars, as they become available) can be used for the prevention of treatment-related febrile neutropenia. The choice of agent depends on convenience, cost, and clinical situation.

For more detailed guidance on managing toxicity, see the Merck Events of Clinical Interest Guidance Document regarding Pembrolizumab.

5.2.2 Treatment after 1st Radiologic Evidence of Disease Progression

If a patient demonstrates progression of disease on CT prior to cycle 3 of treatment (after receiving 2 cycles of cisplatin and gemcitabine), the patient will be considered refractory to chemotherapy. Cisplatin and gemcitabine will not be administered for further cycles and the patient may continue on the trial and receive single agent pembrolizumab.

Immunotherapeutic agents such as pembrolizumab may produce anti-tumor effects by potentiating endogenous cancer-specific immune responses. The response patterns seen with such an approach can manifest as a clinical response after an initial increase in tumor burden or even the appearance of new lesions.

After the site has assessed the first radiographic evidence of progressive disease (PD) by RECIST 1.1 after the addition of pembrolizumab to trial therapy (cycle 3 and beyond), tumor assessment may be repeated ≥ 4 weeks later to confirm PD by iRECIST with the option for continuing treatment while awaiting radiologic confirmation of disease progression.

Subjects may continue to receive study treatment while awaiting confirmation of PD if they are clinically stable as defined by the following criteria:

- Absence of signs and symptoms (including worsening of lab values) indicating disease progression
- No decline in ECOG performance status
- Absence of rapid progression of disease
- Absence of progressive tumor at critical anatomic sites (e.g., cord compression) requiring urgent alternative medical intervention

If repeat imaging shows SD, PR, or CR by iRECIST, the subject may continue on study treatment and undergo regularly scheduled imaging assessments.

Note: If a subject with confirmed radiographic progression (i.e. 2 tumor images at least 28 days apart demonstrating progressive disease) is clinically stable or clinically improved, and there is no further increase in the tumor dimensions at the confirmatory tumor image, an exception may be considered to continue treatment upon consultation with the PI.

5.2.3 Timing of Dose Administration

Trial treatment should be administered on Day 1 of each cycle after all procedures/assessments have been completed as detailed on the Trial Flow Chart (Section 6.0).

The administration of day 1 treatment may be adjusted +/- 3 days to accommodate schedule changes. The administration of day 8 treatment may be adjusted +/- 1 day to accommodate schedule changes.

All trial treatments will be administered on an outpatient basis.

Gemcitabine 750 mg/m^2 and cisplatin 30 mg/m^2 will be given on day 1 and day 8 of a 21-day treatment cycle for a total of 6 cycles. To decrease the risk of hematologic toxicity, gemcitabine will be given prior to cisplatin. Antiemetic agents may include, but are not limited to ondansetron, granisetron, Ativan,

metoclopramide, prochlorperazine, fosaprepitant, aloxi, sancuso and akynzeo. Dexamethasone will not be administered as a premedication in the initial treatment cycle, but may be added at a dose of 10 or 20 mg IV as a premedication if the patient experiences nausea on the regimen. Patients will receive hydration with at least 1 liter of fluid prior to chemotherapy. Hydration will then continue as clinically indicated. A window of -10 minutes and +10 minutes is permitted for chemotherapy administration. Gemcitabine 750 mg/m^2 will be administered IV over 30 minutes (+/- 10 minutes). Cisplatin 30 mg/m^2 will be administered IV over 60 minutes (+/- 10 minutes).

Pembrolizumab 200 mg will be administered as a 30 minute IV infusion every 3 weeks on day 1 after gemcitabine and cisplatin starting with treatment cycle 3. After completion of cycle 6, pembrolizumab will be administered as a single-agent for maintenance therapy every 21 days for up to two years or the subject meets withdrawal criteria.

Every effort should be made to target pembrolizumab infusion timing to be as close to 30 minutes as possible. However, given the variability of infusion pumps from site to site, a window of -5 minutes and +10 minutes is permitted (i.e., infusion time is 30 minutes: -5 min/+10 min. The Pharmacy Manual contains specific instructions for the preparation of the pembrolizumab infusion fluid and administration of infusion solution.

5.2.4 Trial Blinding/Masking

This is an open-label trial; therefore, Merck, investigator, and subject will know the treatment administered.

5.3 Randomization or Treatment Allocation

This is a single-arm study. No randomization will occur.

5.4 Stratification

Patients will be stratified based on number of prior chemotherapeutic regimens (1 versus more than 1; prior cis/gem versus no prior cis/gem).

5.5 Concomitant Medications/Vaccinations (allowed & prohibited)

Medications or vaccinations specifically prohibited in the exclusion criteria are not allowed during the ongoing trial. If there is a clinical indication for one of these or other medications or vaccinations specifically prohibited during the trial, discontinuation from trial therapy or vaccination may be required. The investigator should discuss any questions regarding this with the Merck Clinical team. The final decision on any supportive therapy or vaccination rests with the investigator and/or the subject's primary physician.

5.5.1 Acceptable Concomitant Medications

All treatments that the investigator considers necessary for a subject’s welfare may be administered at the discretion of the investigator in keeping with the community standards of medical care. All concomitant medication will be recorded on the case report form (CRF) including all prescription, over-the-counter (OTC), herbal supplements, and IV medications and fluids. If changes occur during the trial period, documentation of drug dosage, frequency, route, and date may also be included on the CRF.

All concomitant medications received within 28 days before the first dose of trial treatment and 30 days after the last dose of trial treatment should be recorded. Concomitant medications administered after 30 days after the last dose of trial treatment should be recorded for SAEs and ECIs as defined in Section 7.2.

5.5.2 Prohibited Concomitant Medications

Subjects are prohibited from receiving the following therapies during the Screening and Treatment Phase (including retreatment for post-complete response relapse) of this trial:

- Antineoplastic systemic chemotherapy or biological therapy not specified in this protocol
- Immunotherapy not specified in this protocol
- Chemotherapy not specified in this protocol
- Investigational agents other than pembrolizumab
- Radiation therapy
    - Note: Radiation therapy to a symptomatic solitary lesion may be allowed at the investigator's discretion.
- Live vaccines within 30 days prior to the first dose of trial treatment and while participating in the trial. Examples of live vaccines include, but are not limited to, the following: measles, mumps, rubella, varicella/zoster, yellow fever, rabies, BCG, and typhoid vaccine.
- Systemic glucocorticoids for any purpose other than to modulate symptoms from an event of clinical interest of suspected immunologic etiology. An exception can be made to allow for the use of decadon 10 or 20 mg IV as an antiemetic premedication prior to chemotherapy (section 5.2.3). Otherwise, the use of physiologic doses of corticosteroids may be approved after consultation with Merck.

Subjects who, in the assessment by the investigator, require the use of any of the aforementioned treatments for clinical management should be removed from the trial. Subjects may receive other medications that the investigator deems to be medically necessary.

The Exclusion Criteria describes other medications which are prohibited in this trial.

There are no prohibited therapies during the Post-Treatment Follow-up Phase.

5.6 Rescue Medications & Supportive Care

5.6.1 Supportive Care Guidelines for pembrolizumab

Subjects should receive appropriate supportive care measures as deemed necessary by the treating investigator. Suggested supportive care measures for the management of adverse events with potential immunologic etiology are outlined below and in greater detail in the ECI guidance document. Where appropriate, these guidelines include the use of oral or intravenous treatment with corticosteroids as well as additional anti-inflammatory agents if symptoms do not improve with administration of corticosteroids. Note that several courses of steroid tapering may be necessary as symptoms may worsen when the steroid dose is decreased. For each disorder, attempts should be made to rule out other causes such as metastatic disease or bacterial or viral infection, which might require additional supportive care. The treatment guidelines are intended to be applied when the investigator determines the events to be related to pembrolizumab.

Note: if after the evaluation the event is determined not to be related, the investigator is instructed to follow the ECI reporting guidance but does not need to follow the treatment guidance (as outlined in the ECI guidance document). Refer to Section 5.2.1 for dose modification.

It may be necessary to perform conditional procedures such as bronchoscopy, endoscopy, or skin photography as part of evaluation of the event. Suggested conditional procedures, as appropriate, can be found in the ECI guidance document.

5.6.2 Suggested supportive care measures:

5.6.2.1 Pneumonitis:

- For **Grade 2 events**, treat with systemic corticosteroids. When symptoms improve to Grade 1 or less, steroid taper should be started and continued over no less than 4 weeks.
  - Pembrolizumab can be restarted when toxicity resolved to grade 0 or 1. Pembrolizumab should be discontinued if toxicity does not resolve within 12 weeks of last dose or inability to reduce corticosteroid to 10 mg or less of prednisone or equivalent per day within 12 weeks.
- For **Grade 3-4 events**, immediately treat with intravenous steroids. Administer additional anti-inflammatory measures, as needed.
  - Permanently discontinue pembrolizumab.
- Add prophylactic antibiotics for opportunistic infections in the case of prolonged steroid administration.

5.6.2.2 Diarrhea/Colitis:

Subjects should be carefully monitored for signs and symptoms of enterocolitis (such as diarrhea, abdominal pain, blood or mucus in stool, with or without fever) and of bowel perforation (such as peritoneal signs and ileus).

- All subjects who experience diarrhea/colitis should be advised to drink liberal quantities of clear fluids. If sufficient oral fluid intake is not feasible, fluid and electrolytes should be substituted via IV infusion. For Grade 2 or higher diarrhea, consider GI consultation and endoscopy to confirm or rule out colitis.
- For **Grade 2 diarrhea/colitis** that persists greater than 3 days, administer oral corticosteroids.
- For **Grade 3 or 4 diarrhea/colitis** that persists > 1 week, treat with intravenous steroids followed by high dose oral steroids.
- Restart pembrolizumab when grade 2 or 3 toxicity resolved to grade 0 or 1.
- For **grade 2 or 3 diarrhea/colitis** that does not resolve within 12 weeks of last dose or inability to reduce corticosteroid to 10 mg or less of prednisone or equivalent per day within 12 weeks, pembrolizumab should be discontinued.
- For **grade 4 diarrhea/colitis**, pembrolizumab should be permanently discontinued.
- When symptoms improve to Grade 1 or less, steroid taper should be started and continued over no less than 4 weeks.

5.6.2.3 Type 1 diabetes mellitus (if new onset, including diabetic ketoacidosis [DKA]) or ≥ Grade 3 Hyperglycemia, if associated with ketosis (ketonuria) or metabolic acidosis (DKA)

- For **T1DM** or **Grade 3-4** Hyperglycemia
  - Insulin replacement therapy is recommended for Type I diabetes mellitus and for Grade 3-4 hyperglycemia associated with metabolic acidosis or ketonuria.
  - Evaluate patients with serum glucose and a metabolic panel, urine ketones, glycosylated hemoglobin, and C-peptide.
  - Hold pembrolizumab for new onset Type 1 diabetes mellitus or Grade 3-4 hyperglycemia associated with evidence of beta cell failure.
  - Resume pembrolizumab when patients are clinically and metabolically stable.

5.6.2.4 Hypophysitis:

- For **Grade 2** events, treat with corticosteroids. When symptoms improve to Grade 1 or less, steroid taper should be started and continued over no less than 4 weeks. Replacement of appropriate hormones may be required as the steroid dose is tapered.
  - Restart pembrolizumab when toxicity resolved to Grade 0 or 1.
  - If toxicity does not resolve within 12 weeks of last dose or there is inability to reduce corticosteroid to 10 mg or less of prednisone or equivalent per day within 12 weeks, then discontinue pembrolizumab.
- For **Grade 3-4** events, treat with an initial dose of IV corticosteroids followed by oral corticosteroids. When symptoms improve to Grade 1 or less, steroid taper should be started and continued over no less than 4 weeks. Replacement of appropriate hormones may be required as the steroid dose is tapered.
  - For grade 3 toxicity, restart pembrolizumab when toxicity resolved to Grade 0 or 1.
  - If grade 3 toxicity does not resolve within 12 weeks of last dose or there is inability to reduce corticosteroid to 10 mg or less of prednisone or equivalent per day within 12 weeks, then discontinue pembrolizumab.
  - For grade 4 toxicity, permanently discontinue pembrolizumab

5.6.2.5 Hyperthyroidism or Hypothyroidism:

Thyroid disorders can occur at any time during treatment. Monitor patients for changes in thyroid function (at the start of treatment, periodically during treatment, and as indicated based on clinical evaluation) and for clinical signs and symptoms of thyroid disorders.

- **Grade 2** hyperthyroidism events (and **Grade 3-4** hypothyroidism):
  - In hyperthyroidism, non-selective beta-blockers (e.g. propranolol) are suggested as initial therapy.
  - In hypothyroidism, thyroid hormone replacement therapy, with levothyroxine or liothyroinine, is indicated per standard of care.
  - Therapy with pembrolizumab can be continued while treatment for the thyroid disorder is instituted.
- **Grade 3-4** hyperthyroidism
  - Treat with an initial dose of IV corticosteroid followed by oral corticosteroids. When symptoms improve to Grade 1 or less, steroid taper should be started and continued over no less than 4 weeks. Replacement of appropriate hormones may be required as the steroid dose is tapered.
  - For grade 3 hyperthyroidism, restart pembrolizumab when toxicity resolves to grade 0 or 1. If toxicity does not resolve within 12 weeks of last dose or there is inability to reduce corticosteroid to 10 mg or less of prednisone or equivalent per day within 12 weeks, discontinue pembrolizumab.
  - For grade 4 hyperthyroidism, permanently discontinue pembrolizumab

5.6.2.6 Hepatic:

- For **Grade 2** events, monitor liver function tests more frequently until returned to baseline values (consider weekly).
  - Treat with IV or oral corticosteroids
  - Restart pembrolizumab when grade 2 toxicity resolves to grade 0 or 1.
  - Discontinue pembrolizumab if grade 2 toxicity does not resolve within 13 weeks of last dose.

        - For patients with liver metastasis who begin treatment with Grade 2 AST or ALT, if AST or ALT increases by greater than or equal to 50% relative to baseline and lasts for at least 1 week and then patients should be discontinued.
    - For **Grade 3-4** events, treat with intravenous corticosteroids for 24 to 48 hours.
        - For grade 3-4 toxicity, permanently discontinue pembrolizumab.
    - When symptoms improve to Grade 1 or less, a steroid taper should be started and continued over no less than 4 weeks.

5.6.2.7 Renal Failure or Nephritis:

    - For **Grade 2** events, treat with corticosteroids.
        - Restart pembrolizumab when toxicity resolves to grade 0 or 1.
        - If toxicity does not resolve within 12 weeks of last dose or there is inability to reduce corticosteroid to 10 mg or less of prednisone or equivalent per day within 12 weeks, discontinue pembrolizumab.
    - For **Grade 3-4** events, treat with systemic corticosteroids.
        - For grade 3 and 4 events, permanently discontinue pembrolizumab.
    - When symptoms improve to Grade 1 or less, steroid taper should be started and continued over no less than 4 weeks.

- **Management of Infusion Reactions:**
    - Signs and symptoms usually develop during or shortly after drug infusion and generally resolve completely within 24 hours of completion of infusion.
    - For grade 3 or 4 infusion reaction, permanently discontinue pembrolizumab.

Table 4 below shows treatment guidelines for subjects who experience an infusion reaction associated with administration of pembrolizumab (MK-3475).

Table 4 Infusion Reaction Treatment Guidelines

NCI CTCAE Grade	Treatment	Premedication at subsequent dosing
Grade 1<br>Mild reaction; infusion interruption not indicated; intervention not indicated	Increase monitoring of vital signs as medically indicated until the subject is deemed medically stable in the opinion of the investigator.	None
Grade 2<br>Requires infusion interruption but responds promptly to symptomatic treatment (e.g., antihistamines, NSAIDS, narcotics, IV fluids); prophylactic medications indicated for < =24 hrs	**Stop Infusion and monitor symptoms.**<br>Additional appropriate medical therapy may include but is not limited to:<br>IV fluids<br>Antihistamines<br>NSAIDS<br>Acetaminophen<br>Narcotics	Subject may be premedicated 1.5h (± 30 minutes) prior to infusion of pembrolizumab (MK-3475) with:<br><br>Diphenhydramine 50 mg PO (or equivalent dose of antihistamine).

NCI CTCAE Grade	Treatment	Premedication at subsequent dosing
	Increase monitoring of vital signs as medically indicated until the subject is deemed medically stable in the opinion of the investigator.<br>If symptoms resolve within one hour of stopping drug infusion, the infusion may be restarted at 50% of the original infusion rate (e.g., from 100 mL/hr to 50 mL/hr). Otherwise dosing will be held until symptoms resolve and the subject should be premedicated for the next scheduled dose.<br>**Subjects who develop Grade 2 toxicity despite adequate premedication should be permanently discontinued from further trial treatment administration.**	Acetaminophen 500-1000 mg PO (or equivalent dose of antipyretic).
<u>Grades 3 or 4</u><br><br>Grade 3:<br>Prolonged (i.e., not rapidly responsive to symptomatic medication and/or brief interruption of infusion); recurrence of symptoms following initial improvement; hospitalization indicated for other clinical sequelae (e.g., renal impairment, pulmonary infiltrates)<br><br>Grade 4:<br>Life-threatening; pressor or ventilatory support indicated	**Stop Infusion.**<br>Additional appropriate medical therapy may include but is not limited to:<br>IV fluids<br>Antihistamines<br>NSAIDS<br>Acetaminophen<br>Narcotics<br>Oxygen<br>Pressors<br>Corticosteroids<br>Epinephrine<br><br>Increase monitoring of vital signs as medically indicated until the subject is deemed medically stable in the opinion of the investigator.<br>Hospitalization may be indicated.<br>**Subject is permanently discontinued from further trial treatment administration.**	No subsequent dosing
Appropriate resuscitation equipment should be available in the room and a physician readily available during the period of drug administration.		

- All other drug-related toxicity
  - Patients with intolerable or persistent grade 2 drug-related adverse event, pembrolizumab may be held at the physician's discretion. Permanently discontinue pembrolizumab for persistent grade 2 adverse reactions which do not recover to grade 0 or 1 within 12 weeks of the last dose.

- For grade 3 or severe toxicity, hold pembrolizumab and restart when toxicity resolves to grade 0 or 1. If toxicity does not resolve within 12 weeks of last dose or there is inability to reduce corticosteroid to 10 mg or less of prednisone or equivalent per day within 12 weeks, discontinue pembrolizumab.

- For grade 4 toxicity, permanently discontinue pembrolizumab.

5.7 Diet/Activity/Other Considerations

5.7.1 Diet

Subjects should maintain a normal diet unless modifications are required to manage an AE such as diarrhea, nausea or vomiting.

5.7.2 Contraception

Pembrolizumab may have adverse effects on a fetus in utero. Furthermore, it is not known if pembrolizumab has transient adverse effects on the composition of sperm. Non-pregnant, non-breast-feeding women may be enrolled if they are willing to use 2 methods of birth control or are considered highly unlikely to conceive. Highly unlikely to conceive is defined as 1) surgically sterilized, or 2) postmenopausal (a woman who is ≥45 years of age and has not had menses for greater than 1 year will be considered postmenopausal), or 3) not heterosexually active for the duration of the study. The two birth control methods can be either two barrier methods or a barrier method plus a hormonal method to prevent pregnancy. Subjects should start using birth control from study Visit 1 throughout the study period up to 120 days after the last dose of study therapy.

The following are considered adequate barrier methods of contraception: diaphragm, condom (by the partner), copper intrauterine device, sponge, or spermicide. Appropriate hormonal contraceptives will include any registered and marketed contraceptive agent that contains an estrogen and/or a progestational agent (including oral, subcutaneous, intrauterine, or intramuscular agents).

Subjects should be informed that taking the study medication may involve unknown risks to the fetus (unborn baby) if pregnancy were to occur during the study. In order to participate in the study they must adhere to the contraception requirement (described above) for the duration of the study and during the follow-up period defined in section 7.2.2-Reporting of Pregnancy and Lactation to the PI and to Merck. If there is any question that a subject will not reliably comply with the requirements for contraception, that subject should not be entered into the study.

5.7.3 Use in Pregnancy

If a subject inadvertently becomes pregnant while on treatment with pembrolizumab, the subject will immediately be removed from the study. The site will contact the subject at least monthly and document the subject's status until the pregnancy has been completed or terminated. The outcome of the pregnancy will be reported to the IRB and to Merck within 10 days of the PI becoming aware of the outcome. If the outcome is a serious adverse experience (e.g., death, abortion, congenital anomaly, or other disabling or life-threatening complication to the mother or newborn), the PI upon learning of the event, will report within 2 working days to Merck.

The study investigator will make every effort to obtain permission to follow the outcome of the pregnancy and report the condition of the fetus or newborn to the IRB.

5.7.4 Use in Nursing Women

It is unknown whether pembrolizumab is excreted in human milk. Since many drugs are excreted in human milk, and because of the potential for serious adverse reactions in the nursing infant, subjects who are breast-feeding are not eligible for enrollment.

5.8 Subject Withdrawal/Discontinuation Criteria

Subjects may withdraw consent at any time for any reason or be dropped from the trial at the discretion of the investigator should any untoward effect occur. In addition, a subject may be withdrawn by the investigator if enrollment into the trial is inappropriate, the trial plan is violated, or for administrative and/or other safety reasons. Specific details regarding discontinuation or withdrawal are provided in Section 7.1.4 – Other Procedures.

A subject must be discontinued from the trial for any of the following reasons:

- The subject or legal representative (such as a parent or legal guardian) withdraws consent.
- Confirmed radiographic disease progression

  Note: For unconfirmed radiographic disease progression, please see Section 5.2.2

  Note: A subject may be granted an exception to continue on treatment with confirmed radiographic progression if clinically stable or clinically improved, please see Section 5.2.2

- Unacceptable adverse experiences as described in Section 5.2.1.2
- Intercurrent illness that prevents further administration of treatment
- Investigator's decision to withdraw the subject
- The subject has a confirmed positive serum pregnancy test
- Noncompliance with trial treatment or procedure requirements
- The subject is lost to follow-up
- Completed trial treatment with maintenance pembrolizumab.
- Administrative reasons

The End of Treatment and Follow-up visit procedures are listed in Section 6 (Protocol Flow Chart) and Section 7.1.5 (Visit Requirements). After the end of treatment, each subject will be followed for 30 days for adverse event monitoring (serious adverse events will be collected for 90 days after the end of treatment as described in Section 7.1.5.3.1).

Subjects who discontinue for reasons other than progressive disease will have post-treatment follow-up for disease status until disease progression, initiating a non-study cancer treatment, withdrawing consent or becoming lost to follow-up.

After documented disease progression each subject will be followed by telephone or at their SOC clinic visits for overall survival until death, withdrawal of consent, or the end of the study, whichever occurs first.

5.8.1 Discontinuation of Study Therapy after CR

Discontinuation of treatment may be considered for subjects who have attained a confirmed CR that have been treated for at least 24 weeks with pembrolizumab and had at least two treatments with pembrolizumab beyond the date when the initial CR was declared. Subjects who then experience radiographic disease progression may be eligible for up to one year of additional treatment with pembrolizumab via the Second Course Phase at the discretion of the investigator if no cancer treatment was administered since the last dose of pembrolizumab, the subject meets the safety parameters listed in the Inclusion/Exclusion criteria, and the trial is open. Subjects will resume therapy at the same dose and schedule at the time of initial discontinuation. Additional details are provided in Section 7.1.5.5.

5.9 Subject Replacement Strategy

If a subject meets withdrawal criteria before receiving any pembrolizumab treatment (before cycle 3) or voluntarily desires to withdraw from the intervention portion of the trial, the subject may be replaced with another one. The subject withdrawing from the study will be asked whether they will provide consent to continue with the subsequent data collection components of the trial.

5.10 Clinical Criteria for Early Trial Termination

Early trial termination will be the result of the criteria specified below:

5.10.1 Quality or quantity of data recording is inaccurate or incomplete

5.10.2 Poor adherence to protocol and regulatory requirements

5.10.3 Incidence or severity of adverse drug reaction in this or other studies indicates a potential health hazard to subjects

5.10.4 Plans to modify or discontinue the development of the study drug

In the event of Merck's decision to no longer supply study drug, ample notification will be provided so that appropriate adjustments to subject treatment can be made.

6.0 TRIAL FLOW CHART

6.1 Study Flow Chart

					Discontinuation and Follow-Up [3]			
**Activity**	**Screening[1]**	**C1, C3, C5 [2]**	**C2, C4, C6 [2]**	**C7-34 [2]**	**Discon-tinuation**	**Safety Visit [11]**	**Follow-up (q9w) [12, 13]**	**Survival (q9-12w) [14]**
Informed consent	X							
Inclusion, Exclusion	X							
Medical History	X							
Medication Review [4]	X	X	X	X	X			
Adverse Event Review [4]	X	X	X	X	X	X	X	
Physical Exam [4, 5]	X	X	X	X	X	X	X	
ECOG Performance Status [4]	X	X	X	X	X			
CBC, CMPL, Magnesium [6]	X	Day 1, Day 8	Day 1, Day 8	X	X			
LDH, uric acid, phosphorus, direct bilirubin, urinalysis [16]	X	X	X	X	X			
PT, PTT, INR, TSH, T4 free, T3 total, research blood [16]	X		X	Even cycles	X			
CA125 [16]	X	X	X	X	X		X	
Urine or Serum HCG, HIV, HBsAg, HCV [15]	X							
Imaging of chest, abdomen, pelvis [7]	X		10-21 days after C2, C4, C6 [7]	10-21 days after every third cycle starting with C9 [7]			Q9 weeks (every 63 days +/- 14 days) [7]	
CT-guided tumor biopsy [8]	X							
QOL questionnaires [9]	X	Before C3, C5		After C8, C17, C34				
Gemcitabine and Cisplatin		Day 1, Day 8	Day 1, Day 8					
Pembrolizumab [10]		C3, C5	C4, C6	C7-34				
Post-study therapy						X	X	X
Survival status						X	X	X

[1] Screening within 28 days of cycle 1 day 1 treatment

[2] For cycle 1 – 6, day 1 treatment may be adjusted +/- 3 days and day 8 treatment may be adjusted +/- 1 day for administrative reasons. For cycle 7 – 17, day 1 treatment may be adjusted +/- 3 days for administrative reasons (Section 7.1.1.9). Dosing interruptions are permitted in the case of medical/surgical events or logistical reasons not related to study therapy (e.g., elective surgery, unrelated medical events, patient vacation and/or holidays). Subjects should be placed back on study therapy within 3 weeks for he scheduled interruption, unless otherwise discussed with the PI. The reason for interruption should be documented in the patient's study record (Section 5.2.1.2).

[3] Discontinuation and follow-up visits are required only if patient received pembrolizumab

[4] To be performed up to 7 days prior to day 1 of treatment cycles. Procedures do not need to be repeated if day 1 treatment is delayed or held.

[5] Full physical examination at screening, directed physical exam at subsequent time points. Exam includes weight and vital signs

[6] Laboratory studies may be performed up to 72 hours prior to treatment (Section 7.1.2). Labs on day 8 are required to be drawn only if patient is scheduled to receive cisplatin and gemcitabine treatment.

[7] Imaging of chest, abdomen and pelvis will be done at screening and after each even cycle of treatment with a window of 10-21 days after day 1 of cycle 2, cycle 4 and cycle 6. Imaging of chest, abdomen and pelvis will be done approximately every 9 weeks during the maintenance and follow up phases with a window of 10-21 days after day 1 of every third cycle starting with C9 (C9, C12, C15, etc.). Imaging may be performed earlier than defined above if clinically indicated per PI discretion.

[8] Optional tissue collection will be performed during the screening phase. Collection of archival tissue is not required during the screening phase.

[9] FACT-G during screening phase. FACT-G and FACIT-TS-G at other study time points (between cycles 2 – 3, between cycles 4 – 5, between cycles 8 – 9, after cycle 17, after cycle 34).

[10] Pembrolizumab will be administered starting with cycle 3 of therapy.

[11] After end of treatment, subjects will be followed for 30 days for adverse event monitoring. Serious adverse events will be collected for 90 days after the end of treatment (Section 5.8). Subjects with an AE of Grade > 1 will be followed until the resolution of the AE to Grade 0-1 or until the beginning of a new anti-neoplastic therapy, whichever occurs first (Section 7.1.5.3.1). If a subject initiates new anti-cancer therapy within 30 days after the last dose of trial treatment, the 30-day safely follow-up visit must occur before the first dose of the new therapy. Once new anti-cancer therapy has been initiated, the subject will move into survival follow-up (Section 7.1.1.5.3).

[12] Discontinuation of treatment may be considered for subjects who have attained a confirmed CR that have been treated for at least 24 weeks with pembrolizumab and had at least 2 treatments with pembrolizumab beyond the date when initial CR was declared. Subjects who then experience radiographic progression are eligible for up to one year of additional treatment with pembrolizumab via the Second Course Phase at the discretion of the investigator if no cancer treatment was administered since the last dose of pembrolizumab, the subject meets the safety parameters listed in the Inclusion/Exclusion criteria and the trial is open (Section 5.8.1, Section 7.1.5.5). Laboratory tests for screening or entry into the Second Course Phase should be performed within 10 days prior to the first dose of treatment.

[13] After study discontinuation for reasons other than progressive disease, subjects will be followed every 9 weeks for disease status until progression, initiation of non-study cancer treatment, withdrawal of consent or becoming lost to follow-up (section 5.8, Section 7.1.5.4).

[14] After study discontinuation for progression, subjects will be followed by telephone or at clinic visits every 9 – 12 weeks for overall survival status until death, withdrawal of consent, or the end of the study, whichever occurs first (Section 5.8, Section 7.1.5.4.1).

[15] Patients who have had prior hysterectomy and/or bilateral oophorectomy are not required to have a pregnancy test (Urine or Serum HCG).

[16] Laboratory studies may be performed up to 72 hours prior to treatment (Section 7.1.2). Labs do not need to be repeated if day 1 treatment is delayed or held unless clinically indicated per PI.

7.0 TRIAL PROCEDURES

7.1 Trial Procedures

The Trial Flow Chart - Section 6.0 summarizes the trial procedures to be performed at each visit. Individual trial procedures are described in detail below. It may be necessary to perform these procedures at unscheduled time points if deemed clinically necessary by the investigator.

Furthermore, additional evaluations/testing may be deemed necessary by the investigator and/or Merck for reasons related to subject safety. In some cases, such evaluation/testing may be potentially sensitive in nature (e.g., HIV, Hepatitis C, etc.), and thus local regulations may require that additional informed consent be obtained from the subject. In these cases, such evaluations/testing will be performed in accordance with those regulations.

7.1.1 Administrative Procedures

7.1.1.1 Informed Consent

Subjects will be asked to sign and date the Informed Consent and HIPAA Authorization form after receiving a complete explanation of the research study including risks, benefits, and alternatives to participation. Eligible subjects must have the ability to understand and the willingness to sign a written informed consent document.

7.1.1.2 Inclusion/Exclusion Criteria

All inclusion and exclusion criteria will be reviewed by the investigator or qualified designee to ensure that the subject qualifies for the trial.

7.1.1.3 Medical History

A medical history will be obtained by the investigator or qualified designee. Medical history will include all active conditions, and any condition diagnosed within the prior 10 years that are considered to be clinically significant by the Investigator. Details regarding the disease for which the subject has enrolled in this study will be recorded separately and not listed as medical history.

7.1.1.4 Prior and Concomitant Medications Review

7.1.1.4.1 Prior Medications

The investigator or qualified designee will review prior medication use and record prior medication taken by the subject within 28 days before starting the trial. Treatment for the disease for which the subject has enrolled in this study will be recorded separately and not listed as a prior medication.

7.1.1.4.2 Concomitant Medications

The investigator or qualified designee will record medication, if any, taken by the subject during the trial. All medications related to reportable SAEs and ECIs should be recorded as defined in Section 7.2.

7.1.1.5 Disease Details and Treatments

7.1.1.5.1 Disease Details

The investigator or qualified designee will obtain prior and current details regarding disease status.

7.1.1.5.2 Prior Treatment Details

The investigator or qualified designee will review all prior cancer treatments including systemic treatments, radiation and surgeries.

7.1.1.5.3 Subsequent Anti-Cancer Therapy Status

The investigator or qualified designee will review all new anti-neoplastic therapy initiated after the last dose of trial treatment. If a subject initiates a new anti-cancer therapy within 30 days after the last dose of trial treatment, the 30-day Safety Follow-up visit must occur before the first dose of the new therapy. Once new anti-cancer therapy has been initiated the subject will move into survival follow-up.

7.1.1.6 Assignment of Subject Number

7.1.1.7 Subjects will be assigned a sequential screening number S001, S002, etc. This screening ID number will no longer be used after screening is complete.

7.1.1.8 Once a screening subject has been determined to meet eligibility criteria and has signed the appropriate consents, the subject will be assigned a 3-digit numeric subject ID that follows the standard SOCCI format (001, 002, 003, etc.).

7.1.1.9 Trial Compliance (Medication/Diet/Activity/Other)

Trial medications will be administered in a chemotherapy infusion area. Day 1 treatments will be scheduled every 21 days +/- 3 days. Day 8 treatments will be schedule with an accommodation of +/- 1 day for schedule changes.

7.1.2 Clinical Procedures/Assessments

During the screening phase, subjects will sign consent and undergo evaluations for inclusion and exclusion criteria, demographics, medical history, medication review, adverse event review, physical examination, vital signs and weight, ECOG performance status, laboratory evaluations (CBC with differential, comprehensive metabolic panel, LDH, uric acid, phosphorus, magnesium, direct bilirubin, urinalysis, coagulation studies, thyroid studies, CA125, urine or serum pregnancy test (if indicated), optional blood for correlative studies), CT scan of the chest, abdomen and pelvis and optional CT-guided biopsy of tumor if possible. Pathology reports from the original surgery will be reviewed to confirm diagnosis of epithelial ovarian, peritoneal or fallopian tube cancer.

During the treatment phase, subjects will be monitored with history and physical examination, vital signs, weight, assessment of adverse events and mediation review prior to each treatment cycle. These assessments may occur during a clinic visit in between cycles that is distinct from a treatment day. Laboratory studies can be performed up to 72 hours prior to treatment. Assessment of thyroid function and blood collection for correlative studies will be performed prior to every even cycle (cycles 2, 4, 6). CT scan of the chest, abdomen and pelvis will be performed after every second cycle during chemotherapy (window of 10-21 days after day 1 of cycles 2, 4, 6) and every 9 weeks thereafter or as clinically indicated (window of 10-21 days after day 1 of cycles 9, 12, 15, etc.). Similar assessments

will occur prior to each cycle of treatment when the subject enters the maintenance phase. Clinically significant labs will be repeated within 3 days.

7.1.2.1 Adverse Event (AE) Monitoring

The investigator or qualified designee will assess each subject to evaluate for potential new or worsening AEs as specified in the Trial Flow Chart and more frequently if clinically indicated. Adverse experiences will be graded and recorded throughout the study and during the follow-up period according to NCI CTCAE Version 4.0 (see Section 11.2). Toxicities will be characterized in terms regarding seriousness, causality, toxicity grading, and action taken with regard to trial treatment.

For subjects receiving treatment with pembrolizumab all AEs of unknown etiology associated with pembrolizumab exposure should be evaluated to determine if it is possibly an event of clinical interest (ECI) of a potentially immunologic etiology (termed immune-related adverse events, or irAEs); see the separate ECI guidance document regarding the identification, evaluation and management of potential irAEs.

Please refer to section 7.2 for detailed information regarding the assessment and recording of AEs.

7.1.2.2 Full Physical Exam

The investigator or qualified designee will perform a complete physical exam during the screening period. Clinically significant abnormal findings should be recorded as medical history. A full physical exam should be performed during screening.

7.1.2.3 Directed Physical Exam

For cycles that do not require a full physical exam per the Trial Flow Chart, the investigator or qualified designee will perform a directed physical exam as clinically indicated prior to trial treatment administration either on the date of treatment or during a clinic visit preceding the date of treatment.

7.1.2.4 Vital Signs

The investigator or qualified designee will take vital signs at screening, prior to the administration of each dose of trial treatment and at treatment discontinuation as specified in the Trial Flow Chart (Section 6.0). Vital signs should include temperature, pulse, respiratory rate, weight and blood pressure. Height will be measured at screening only.

7.1.2.5 Eastern Cooperative Oncology Group (ECOG) Performance Scale

The investigator or qualified designee will assess ECOG status (see Section 11.1) at screening, prior to the administration of each dose of trial treatment and discontinuation of trial treatment as specified in the Trial Flow Chart.

7.1.2.6 Tumor Imaging and Assessment of Disease

A baseline CT scan of the chest, abdomen and pelvis will be performed within 28 days of initiating treatment and RECIST 1.1 criteria (See Appendix C) will be used to verify eligibility with measurable disease. Patients with allergy to IV contrast will undergo imaging with MRI of the abdomen and pelvis with gadolinium and CT scan of the chest without contrast. Imaging will be performed after every even cycle (10-21 days after day 1 of cycles 2, 4, 6) during chemotherapy and every 3 cycles (10-21 days after day 1 of cycles 9, 12, 15, etc.) thereafter, or earlier if clinically indicated to assess for response.

7.1.2.7 Tumor Tissue Collection and Correlative Studies Blood Sampling

If subject provides optional consent for biopsy, CT-guided biopsy of recurrent tumor will be obtained prior to initiating treatment. Attempts will be made to obtain archival formalin-fixed, paraffin-embedded specimens from the original cytoreductive surgery. Collection of archival tissue is not required during the screening phase. Qualtek will provide tissue shipping instructions.

If subject provides optional consent for blood collection, blood will be collected at baseline and thereafter prior to every even cycle of treatment and will be stored for future correlative studies.

7.1.3 Laboratory Procedures/Assessments

Details regarding specific laboratory procedures/assessments to be performed in this trial are provided below. Laboratory Safety Evaluations (Hematology, Chemistry and Urinalysis)

Laboratory tests for hematology, chemistry, urinalysis, and others are specified in Table 5.

Table 5 Laboratory Tests

Hematology	Chemistry	Urinalysis	Other
Hematocrit	Albumin	Blood	Serum β-human chorionic gonadotropin†
Hemoglobin	Alkaline phosphatase	Glucose	(β-hCG)†
Platelet count	Alanine aminotransferase (ALT)	Protein	PT (INR)
WBC (total and differential)	Aspartate aminotransferase (AST)	Specific gravity	aPTT
Red Blood Cell Count	Lactate dehydrogenase (LDH)	Microscopic exam *(If abnormal)*	Total thriiodothyronine (T3)
Absolute Neutrophil Count	Carbon Dioxide ‡	results are noted	Free thyroxine (T4)
Absolute Lymphocyte Count	*(CO_2 or bicarbonate)*	Urine pregnancy test †	Thyroid stimulating hormone (TSH)
	Uric Acid		
	Calcium		
	Chloride		Blood for correlative studies (optional)
	Glucose		
	Phosphorus		
	Potassium		
	Sodium		
	Magnesium		
	Total Bilirubin		
	Direct Bilirubin *(If total bilirubin is elevated above the upper limit of normal)*		
	Total protein		
	Blood Urea Nitrogen		

† Perform on women of childbearing potential only. If urine pregnancy results cannot be confirmed as negative, a serum pregnancy test will be required.

‡ If considered standard of care in your region.

Laboratory tests for screening or entry into the Second Course Phase should be performed within 10 days prior to the first dose of treatment. After Cycle 1, pre-dose laboratory procedures can be conducted up to 72 hours prior to dosing. Results must be reviewed by the investigator or qualified designee and found to be acceptable prior to each dose of trial treatment. If review is found to be acceptable, the investigator or qualified designee will enter an order to release treatment (no other specific documentation will be required).

7.1.3.1 Pharmacokinetic/Pharmacodynamic Evaluations

7.1.3.1.1 Blood Collection for Serum Pembrolizumab

7.1.3.1.2 Blood Collection for Anti-Pembrolizumab Antibodies

Sample collection and storage instructions for blood samples will be provided in a separate document and will follow institutional guidelines.

7.1.4 Other Procedures

7.1.4.1 Quality of Life Questionnaires

FACT-G and FACIT-TS-G are patient self-administered questionnaires. FACT-G will be collected at Screening. FACT-G and FACIT-TS-G will be collected between Cycle 2 - 3, between Cycle 4- 5, and if applicable between cycle 8 - 9 (approximately and 6 months since Cycle 1 Day 1) after cycle 17 (approximately 12 months after Cycle 1 Day 1) and after cycle 34 (approximately 24 months after Cycle 1 Day 1).

7.1.4.2 Withdrawal/Discontinuation

When a subject discontinues/withdraws prior to trial completion, all applicable activities scheduled for the final trial visit should be performed at the time of discontinuation. Any adverse events which are present at the time of discontinuation/withdrawal should be followed in accordance with the safety requirements outlined in Section 7.2 - Assessing and Recording Adverse Events. Subjects who a) attain a CR or b) complete 12 months of treatment with pembrolizumab may discontinue treatment with the option of restarting treatment if they meet the criteria specified in Section 7.1.5.5. After discontinuing treatment following assessment of CR, these subjects should return to the site for a Safety Follow-up Visit (described in Section 7.1.5.3.1) and then proceed to the Follow-Up Period of the study (described in Section 7.1.5.4).

7.1.4.3 Blinding/Unblinding

Not applicable.

7.1.5 Visit Requirements

Visit requirements are outlined in Section 6.0 - Trial Flow Chart. Specific procedure-related details are provided above in Section 7.1 - Trial Procedures.

7.1.5.1 Screening

7.1.5.1.1 Screening Period

Screening procedures are indicated in the trial flow chart (6.0) and should occur within 28 days of initiating treatment.

7.1.5.2 Treatment Period

Subjects will receive six cycles of chemotherapy (or less than 6 if indicated to be held or discontinued per protocol). Pembrolizumab will be added during cycle 3 and will be administered as maintenance therapy every 21 days after the completion of chemotherapy. Maintenance therapy will continue for up to an additional 28 cycles until disease progression or the subject meets withdrawal criteria.

7.1.5.3 Post-Treatment Visits

7.1.5.3.1 Safety Follow-Up Visit

The mandatory Safety Follow-Up Visit should be conducted approximately 30 days after the last dose of trial treatment or before the initiation of a new anti-cancer treatment, whichever comes first. All AEs that occur prior to the Safety Follow-Up Visit should be recorded. Subjects with an AE of Grade > 1 will be followed until the resolution of the AE to Grade 0-1 or until the beginning of a new anti-neoplastic therapy, whichever occurs first. SAEs that occur within 90 days of the end of treatment or before initiation of a new anti-cancer treatment should also be followed and recorded. Subjects who are eligible for retreatment with pembrolizumab (as described in Section 7.1.5.5) may have up to two safety follow-up visits, one after the Treatment Period and one after the Second Course Phase.

The safety follow-up visit is only required if subjects received the study agent, pembrolizumab.

7.1.5.4 Follow-up Visits

Subjects who discontinue trial treatment for a reason other than disease progression will move into the Follow-Up Phase and should be assessed every 9 weeks (63 ± 14 days) or as clinically indicated by radiologic imaging to monitor disease status. Every effort should be made to collect information regarding disease status until the start of new anti-neoplastic therapy, disease progression, death, end of the study or if the subject begins retreatment with pembrolizumab as detailed in Section 7.1.5.5. Information regarding post-study anti-neoplastic treatment will be collected if new treatment is initiated.

Subjects who are eligible to receive retreatment with pembrolizumab according to the criteria in Section 7.1.5.5 will move from the follow-up phase to the Second Course Phase when they experience disease progression. Details are provided in Section 6.2 – Trial Flow Chart for Retreatment.

Follow-up visits are only required if subjects received the study agent, pembrolizumab.

7.1.5.4.1 Survival Follow-up

Once a subject experiences confirmed disease progression or starts a new anti-cancer therapy, the subject moves into the survival follow-up phase and should be assessed (in clinic or contacted by telephone) every 9-12 weeks to assess for survival status until death, withdrawal of consent, or the end of the study, whichever occurs first.

7.1.5.5 Second Course Phase (Retreatment Period)

Subjects who stop pembrolizumab with SD, PR or CR may be eligible for up to one year of additional pembrolizumab therapy if they progress after stopping study treatment. This retreatment is termed the Second Course Phase of this study and is only available if the study remains open and the subject meets the following conditions:

- **Either**
    - Stopped initial treatment with pembrolizumab after attaining an investigator-determined confirmed CR according to RECIST 1.1, and

- Was treated for at least 24 weeks with pembrolizumab before discontinuing therapy
- Received at least two treatments with pembrolizumab beyond the date when the initial CR was declared

OR

- Had SD, PR or CR and stopped pembrolizumab treatment after 24 months of study therapy for reasons other than disease progression or intolerability

AND

- Experienced an investigator-determined confirmed radiographic disease progression after stopping their initial treatment with pembrolizumab
- Did not receive any anti-cancer treatment since the last dose of pembrolizumab
- Has a performance status of 0 or 1 on the ECOG Performance Scale
- Demonstrates adequate organ function as detailed in Section 5.1.2
- Female subject of childbearing potential should have a negative serum or urine pregnancy test within 72 hours prior to receiving retreatment with study medication.
- Female subject of childbearing potential should be willing to use 2 methods of birth control or be surgically sterile, or abstain from heterosexual activity for the course of the study through 120 days after the last dose of study medication (Reference Section 5.7.2). Subjects of child bearing potential are those who have not been surgically sterilized or have been free from menses for > 1 year.
- Does not have a history or current evidence of any condition, therapy, or laboratory abnormality that might interfere with the subject's participation for the full duration of the trial or is not in the best interest of the subject to participate, in the opinion of the treating investigator.

Subjects who restart treatment will be retreated at the same dose and dose interval as when they last received pembrolizumab. Treatment will be administered for up to one additional year.

Visit requirements are outlined in Section 6.0 – Trial Flow Chart.

7.2 Assessing and Recording Adverse Events

7.2.1 Definition of Adverse Event

An adverse event is defined as any untoward medical occurrence in a patient or clinical investigation subject administered a pharmaceutical product and which does not necessarily have to have a causal relationship with this treatment. An adverse event can therefore be any unfavorable and unintended sign (including an abnormal laboratory finding, for example), symptom, or disease temporally associated with the use of a medicinal product or protocol-specified procedure, whether or not considered related to the medicinal product or protocol-specified procedure. Any worsening (i.e., any clinically significant adverse change in frequency and/or intensity) of a preexisting condition that is temporally associated with the use of Merck's product, is also an adverse event.

Changes resulting from normal growth and development that do not vary significantly in frequency or severity from expected levels are not to be considered adverse events. Examples of this may include, but are not limited to, teething, typical crying in infants and children and onset of menses or menopause occurring at a physiologically appropriate time.

Merck's product includes any pharmaceutical product, biological product, device, diagnostic agent or protocol-specified procedure, whether investigational (including placebo or active comparator medication) or marketed, manufactured by, licensed by, provided by or distributed by Merck for human use.

Adverse events may occur during the course of the use of Merck's product in clinical trials or within the follow-up period specified by the protocol, or prescribed in clinical practice, from overdose (whether accidental or intentional), from abuse and from withdrawal.

Adverse events may also occur in screened subjects during any pre-allocation baseline period as a result of a protocol-specified intervention, including washout or discontinuation of usual therapy, diet, placebo treatment or a procedure.

Progression of the cancer under study is not considered an adverse event unless it is considered to be drug related by the investigator.

All adverse events with the exception of grade 1 or 2 laboratory abnormalities will be recorded from the time the consent form is signed through 30 days following cessation of treatment and at each examination on the Adverse Event case report forms/worksheets. The reporting timeframe for adverse events meeting any serious criteria is described in section 7.2.3.1.

7.2.2 Severity of Adverse Events

All non-hematologic adverse events will be graded according to the NCI Common Terminology Criteria for Adverse Events (CTCAE) version 4.0. The CTCAE v4 is available at http://ctep.cancer.gov/reporting/ctc.html

If no CTCAE grading is available, the severity of an AE is graded as follows:

Mild (grade 1): the event causes discomfort without disruption of normal daily activities.

Moderate (grade 2): the event causes discomfort that affects normal daily activities.

Severe (grade 3): the event makes the patient unable to perform normal daily activities or significantly affects his/her clinical status.

Life-threatening (grade 4): the patient was at risk of death at the time of the event.

Fatal (grade 5): the event caused death.

7.2.3 Definition of an Overdose for This Protocol and Reporting of Overdose to the IRB and to Merck

For purposes of this trial, an overdose of pembrolizumab will be defined as any dose of 1,000 mg or greater (≥5 times the indicated dose). No specific information is available on the treatment of overdose of pembrolizumab. Appropriate supportive treatment should be provided if clinically indicated. In the event of overdose, the subject should be observed closely for signs of toxicity. Appropriate supportive treatment should be provided if clinically indicated

If an adverse event(s) is associated with ("results from") the overdose of a Merck product, the adverse event(s) is reported as a serious adverse event, even if no other seriousness criteria are met.

If a dose of Merck's product meeting the protocol definition of overdose is taken without any associated clinical symptoms or abnormal laboratory results, the overdose is reported as a non-serious Event of Clinical Interest (ECI), using the terminology "accidental or intentional overdose without adverse effect." As per institutional policies, an overdose is considered an SAE and must be reported (See section 7.2.5)

All reports of overdose with and without an adverse event must be reported within 2 working days to Merck Global Safety. See section on SAE reporting for further information regarding institutional policy for reporting SAEs. (Attn: Worldwide Product Safety; FAX 215 993-1220).

7.2.4 Reporting of Pregnancy and Lactation to the IRB and to Merck

Although pregnancy and lactation are not considered adverse events, it is the responsibility of investigators or their designees to report any pregnancy or lactation in a subject (spontaneously reported to them), that occurs during the trial or within 120 days of completing the trial completing the trial, or 30 days following cessation of treatment if the subject initiates new anticancer therapy, whichever is earlier. All subjects who become pregnant must be followed to the completion/termination of the pregnancy. Pregnancy outcomes of spontaneous abortion, missed abortion, benign hydatidiform mole, blighted ovum, fetal death, intrauterine death, miscarriage and stillbirth must be reported as serious events (Important Medical Events). If the pregnancy continues to term, the outcome (health of infant) must also be reported.

Such events must be reported within 2 working days to Merck Global Safety. (Attn: Worldwide Product Safety; FAX 215 993-1220)

7.2.5 Immediate Reporting of Adverse Events to the IRB and to Merck

7.2.5.1.1 Serious Adverse Events

A serious adverse event is any adverse event occurring at any dose or during any use of Merck's product that:

- Results in death;
- Is life threatening;
- Results in persistent or significant disability/incapacity;
- Results in or prolongs an existing inpatient hospitalization;
- Is a congenital anomaly/birth defect;
- Is a new cancer (that is not a condition of the study);
- Is associated with an overdose;
- Is another important medical event

Refer to Table 6 for additional details regarding each of the above criteria.

Progression of the cancer under study is not considered an adverse event unless it results in hospitalization or death.

Any serious adverse event, or follow up to a serious adverse event, including death due to any cause that occurs to any subject from the time the consent is signed through 90 days following cessation of treatment, or the initiation of new anti-cancer therapy, whichever is earlier, whether or not related to Merck's product, must be reported within 2 working days to Merck Global Safety upon the PI becoming aware of the event.

Non-serious Events of Clinical Interest will be forwarded to Merck Global Safety and will be handled in the same manner as SAEs.

Additionally, any serious adverse event, considered by an investigator who is a qualified physician to be related to Merck's product that is brought to the attention of the investigator at any time outside of the time period specified in the previous paragraph also must be reported immediately to the IRB and to Merck.

SAE reports and any other relevant safety information are to be forwarded to the Merck Global Safety facsimile number: +1-215-993-1220

A copy of all 15 Day Reports and Annual Progress Reports is submitted as required by FDA, European Union (EU), Pharmaceutical and Medical Devices agency (PMDA) or other local regulators. Investigators will cross reference this submission according to local regulations to the Merck Investigational Compound Number (IND, CSA, etc.) at the time of submission. Additionally investigators will submit a copy of these reports to Merck & Co., Inc. (Attn: Worldwide Product Safety; FAX 215 993-1220) at the time of submission to FDA.

All subjects with serious adverse events must be followed up for outcome.

7.2.5.2 Events of Clinical Interest

Selected non-serious and serious adverse events are also known as Events of Clinical Interest (ECI) and must be recorded as such on the Adverse Event case report forms/worksheets and reported within 2 working days to Merck Global Safety (Attn: Worldwide Product Safety; FAX 215 993-1220). Events of clinical interest for this trial include:

1. An overdose of Merck product, as defined in Section 7.2.1 - Definition of an Overdose for This Protocol and Reporting of Overdose to the IRB, that is not associated with clinical symptoms or abnormal laboratory results.

2. An elevated AST or ALT lab value that is greater than or equal to 3X the upper limit of normal and an elevated total bilirubin lab value that is greater than or equal to 2X the upper limit of normal and, at the same time, an alkaline phosphatase lab value that is less than 2X the upper limit of normal, as determined by way of protocol-specified laboratory testing or unscheduled laboratory testing.*

> <u>*Note:</u> These criteria are based upon available regulatory guidance documents. The purpose of the criteria is to specify a threshold of abnormal hepatic tests that may require an additional evaluation for an underlying etiology. The trial site guidance for assessment and follow up of these criteria can be found in the Investigator Trial File Binder (or equivalent).3. Additional adverse events:
>
> A separate guidance document has been provided entitled "Event of Clinical Interest Guidance Document" (previously entitled, "Event of Clinical Interest and Immune-Related Adverse Event Guidance Document"). This document provides guidance regarding identification, evaluation and management of ECIs and irAEs.
>
> ECIs (both non-serious and serious adverse events) identified in this guidance document from the date of first dose through 90 days following cessation of treatment, or 30 days after the initiation of a new anticancer therapy, whichever is earlier, need to be reported within 2 working days to Merck Global Safety. (Attn: Worldwide Product Safety; FAX 215 993-1220), regardless of attribution to study treatment, consistent with standard SAE reporting guidelines.

Subjects should be assessed for possible ECIs prior to each dose. Lab results should be evaluated and subjects should be asked for signs and symptoms suggestive of an immune-related event. Subjects who develop an ECI thought to be immune-related should have additional testing to rule out other etiologic causes. If lab results or symptoms indicate a possible immune-related ECI, then additional testing should be performed to rule out other etiologic causes. If no other cause is found, then it is assumed to be immune-related.

7.2.6 Evaluating Adverse Events

An investigator who is a qualified physician will evaluate all adverse events according to the NCI Common Terminology for Adverse Events (CTCAE), version 4.0. Any adverse event which changes CTCAE grade over the course of a given episode will have each change of grade recorded on the adverse event case report forms/worksheets.

All adverse events regardless of CTCAE grade must also be evaluated for seriousness.

Grade 1 and 2 lab abnormalities will not be collected. The study will only collect grade 3 and 4 lab abnormalities as adverse events.

7.3 Reporting Requirements for Adverse Events

7.3.1 Expedited reporting

The Principal Investigator must be notified by study staff within 24 hours of learning of any serious adverse events, regardless of attribution, occurring during the study or within 30 days of the last administration of the study drug.

Phone number for expedited reporting: Christine Walsh, Phone number: 310-423-5456

7.3.2 Reporting to the Institutional Review Board (IRB)

The CSMC IRB requires that investigators report all adverse events that may represent an unanticipated problem involving risks to subjects or others as defined below. All adverse events (those involving subjects who were enrolled at CSMC), that have a reasonable possibility of relationship to the study AND meet the following criteria must be reported to the IRB:

- Unanticipated (regardless of severity); OR

- Anticipated and serious

All reportable events should be submitted in Webridge to the Office of Research Compliance and Quality Improvement as soon as possible, but no more than **10 days from the investigator's** awareness of the event. The report must contain at least:

- Identification of the PI, study coordinator (if applicable), contact information, study title, and IRB number.

- A detailed summary of the problem, including all relevant details and the PI's assessment of the events leading up to the problem, to assist the IRB in its evaluation.

- A description of any action taken to address or remedy the problem, including a description of the resolution, if any, or current status.

• An assessment as to whether any changes are required in the conduct of the research to resolve the problem or prevent further problems.

Table 6 Evaluating Adverse Events

An investigator, who is a qualified physician, will evaluate all adverse events as to:

**V4.0 CTCAE Grading**	**Grade 1**	**Mild; asymptomatic or mid symptoms; clinical or diagnostic observations only; intervention not indicated.**
	**Grade 2**	**Moderate; minimal, local or noninvasive intervention indicated; limiting age-appropriate instrumental ADL.**
	**Grade 3**	**Severe or medically significant but not immediately life-threatening; hospitalization or prolongation or hospitalization indicated; disabling; limiting self-care ADL.**
	**Grade 4**	**Life threatening consequences; urgent intervention indicated.**
	**Grade 5**	**Death related to AE**
**Seriousness**	A serious adverse event is any adverse event occurring at any dose or during any use of Sponsor product that:	
	†**Results in death**; or	
	†**Is life threatening; or** places the subject, in the view of the investigator, at immediate risk of death from the event as it occurred (Note: This does not include an adverse event that, had it occurred in a more severe form, might have caused death.); or	
	†**Results in a persistent or significant disability/incapacity** (substantial disruption of one's ability to conduct normal life functions); or	
	†**Results in or prolongs an existing inpatient hospitalization** (hospitalization is defined as an inpatient admission, regardless of length of stay, even if the hospitalization is a precautionary measure for continued observation. (Note: Hospitalization [including hospitalization for an elective procedure] for a preexisting condition which has not worsened does not constitute a serious adverse event.); or	
	†**Is a congenital anomaly/birth defect** (in offspring of subject taking the product regardless of time to diagnosis);or	
	**Is a** new **cancer;** (that is not a condition of the study) **or**	
	**Is an overdose** (whether accidental or intentional). Any adverse event associated with an overdose is considered a serious adverse event. An overdose that is not associated with an adverse event is considered a non-serious event of clinical interest and must be reported within 2 business days to Merck.	
	**Other important medical events** that may not result in death, not be life threatening, or not require hospitalization may be considered a serious adverse event when, based upon appropriate medical judgment, the event may jeopardize the subject and may require medical or surgical intervention to prevent one of the outcomes listed previously (designated above by a †).	
**Duration**	Record the start and stop dates of the adverse event. If less than 1 day, indicate the appropriate length of time and units	
**Action taken**	Did the adverse event cause the Sponsor product to be discontinued?	
**Relationship to test drug**	Did the Sponsor product cause the adverse event? The determination of the likelihood that the Sponsor product caused the adverse event will be provided by an investigator who is a qualified physician. The investigator's signed/dated initials on the	

	source document or worksheet that supports the causality noted on the AE form, ensures that a medically qualified assessment of causality was done. This initialed document must be retained for the required regulatory time frame. The criteria below are intended as reference guidelines to assist the investigator in assessing the likelihood of a relationship between the test drug and the adverse event based upon the available information.<br>**The following components are to be used to assess the relationship between the Sponsor product and the AE**; the greater the correlation with the components and their respective elements (in number and/or intensity), the more likely the Sponsor product caused the adverse event (AE):	
	**Exposure**	Is there evidence that the subject was actually exposed to the Sponsor product such as: reliable history, acceptable compliance assessment (pill count, diary, etc.), expected pharmacologic effect, or measurement of drug/metabolite in bodily specimen?
	**Time Course**	Did the AE follow in a reasonable temporal sequence from administration of the Sponsor product?<br>Is the time of onset of the AE compatible with a drug-induced effect (applies to trials with investigational medicinal product)?
	**Likely Cause**	Is the AE not reasonably explained by another etiology such as underlying disease, other drug(s)/vaccine(s), or other host or environmental factors

**Relationship to Sponsor product (continued)**	**The following components are to be used to assess the relationship between the test drug and the AE: (continued)**	
	**Dechallenge**	Was the Sponsor product discontinued or dose/exposure/frequency reduced?<br>If yes, did the AE resolve or improve?<br>If yes, this is a positive dechallenge. If no, this is a negative dechallenge.<br>(Note: This criterion is not applicable if: (1) the AE resulted in death or permanent disability; (2) the AE resolved/improved despite continuation of the Sponsor product; or (3) the trial is a single-dose drug trial); or (4) Sponsor product(s) is/are only used one time.)
	**Rechallenge**	Was the subject re-exposed to the Sponsor product in this study?<br>If yes, did the AE recur or worsen?<br>If yes, this is a positive rechallenge. If no, this is a negative rechallenge.<br>(Note: This criterion is not applicable if: (1) the initial AE resulted in death or permanent disability, or (2) the trial is a single-dose drug trial); or (3) Sponsor product(s) is/are used only one time).<br>NOTE: IF A RECHALLENGE IS PLANNED FOR AN ADVERSE EVENT WHICH WAS SERIOUS AND WHICH MAY HAVE BEEN CAUSED BY THE SPONSOR PRODUCT, OR IF REEXPOSURE TO THE SPONSOR PRODUCT POSES ADDITIONAL POTENTIAL SIGNIFICANT RISK TO THE SUBJECT, THEN THE

		RECHALLENGE MUST BE APPROVED IN ADVANCE BY THE U.S. CLINICAL MONITOR AS PER DOSE MODIFICATION GUIDELINES IN THE PROTOCOL.
	**Consistency with Trial Treatment Profile**	Is the clinical/pathological presentation of the AE consistent with previous knowledge regarding the Sponsor product or drug class pharmacology or toxicology?
The assessment of relationship will be reported on the case report forms /worksheets by an investigator who is a qualified physician according to his/her best clinical judgment, including consideration of the above elements.		
**Record one of the following**		**Use the following scale of criteria as guidance (not all criteria must be present to be indicative of a Sponsor product relationship).**
**Yes, there is a reasonable possibility of Sponsor product relationship.**		There is evidence of exposure to the Sponsor product. The temporal sequence of the AE onset relative to the administration of the Sponsor product is reasonable. The AE is more likely explained by the Sponsor product than by another cause.
**No, there is not a reasonable possibility of Sponsor product relationship**		Subject did not receive the Sponsor product OR temporal sequence of the AE onset relative to administration of the Sponsor product is not reasonable OR there is another obvious cause of the AE. (Also entered for a subject with overdose without an associated AE.)

7.3.3 Investigator Responsibility for Reporting Adverse Events

All Adverse Events will be reported to regulatory authorities, IRB/IECs and to Merck in accordance with all applicable global laws and regulations.

8.0 STATISTICAL ANALYSIS PLAN

8.1 Trial Design

The design for this one-arm phase II trial follows the classical two-stage design proposed by Simon (1989) [31].

The primary efficacy endpoint is overall response rate (ORR) defined as the proportion of patients who achieve a complete or partial response per RECIST 1.1 criteria [32, 33].

- Complete response (CR): disappearance of all target lesions. Any pathological lymph nodes (whether target or non-target) must have reduction in short axis to <10 mm.

- Partial response (PR): at least a 30% decrease in the sum of diameters of target lesions, taking as reference the baseline sum diameters.

- Stable disease (SD): Neither insufficient shrinkage to qualify for PR nor sufficient increase to qualify for PD (see below), taking as reference the smallest sum diameters while on study.

- Progressive disease (PD): at least a 20% increase in the sum of diameters of target lesions, taking as reference the smallest sum on study (this includes the baseline sum if that is the smallest on study). In addition to the relative increase of 20%, the sum must also demonstrate an absolute increase of at least 5 mm. The appearance of one or more new lesions is also considered progression.

Response will be assessed at each time point and the best overall response rate will be determined accounting for response at subsequent time points through the first 6 cycles of therapy [33].

A proportion of patients with a favorable response of less than 11.1% will be of no interest. The new treatment would be of interest if the proportion of patients with response is at least 30.0%. Twenty-five patients would be needed to test the null hypothesis: $p \leq 0.111$ against the alternative hypothesis: $p \geq 0.300$ at the 5.0% level of significance and with 80% power. If the trial progresses until 25 patients are evaluated and 6 or more patients with favorable response are observed then the null hypothesis is rejected.

8.2 Early Stopping for Futility

The early stopping point is 18 patients. If 2 or fewer patients with favorable response are observed when 18 patients are accrued, then the null hypothesis is accepted and the trial is terminated. The probability of early stopping under the null is 0.68 and under the alternative is 0.06.

8.3 Design Operating Characteristics

Hypothesis: H0: p = 0.110669 and H1: p = 0.3

Total sample size = 25 and interim analysis at N=18

P (accept H_0 early| H_0) = 0.6793
P (accept H_0 early| H_1) = 0.0600
P (reject H_0 early| H_0) = 0.0000
P (reject H_0 early| H_1) = 0.0000
P (accept H_0 at end| H_0) = 0.2708
P (accept H_0 at end| H_1) = 0.1397
P (reject H_0 at end| H_0) = 0.0499
P (reject H_0 at end| H_1) = 0.8004
P (early stop| H_0) = 0.6793
P (early stop| H_1) = 0.0600

Overall type I error: 0.0499

Overall power: 0.8004

Average Sample Number (under H0): 20.2451

Average Sample Number (under H1): 24.5803

8.4 Early Stopping for Safety

An undesirable event is defined as a grade 3 or 4 pembrolizumab attributable toxicity that results in permanent discontinuation of the drug (see table 3 and section 5.6.1). Let P_t be the true probability of an undesirable event. The trial will stop if there is statistical evidence that P_t exceeds 0.4. We will use a Bayesian sequential design by checking whether P_t exceeds this threshold value after each patient is evaluable for this event. The decision rule is to stop the trial if the posterior probability that P_t exceeds the threshold is 0.95; for example, P (P_t > 0.4 | data) > 0.95. A noninformative prior distribution for P_t will be used. Tables 7A and 7B give the stopping rules for the design at each look and column 2 gives the maximum number of patients with undesirable events in order for the trial to proceed. For example, in Table 7A, if 13 or more undesirable events are observed after enrolling 18 patients, the trial stops. The third column gives the probability of stopping the trial when in fact, the true P_t = 0.40. This is the equivalent of the Bayesian type I error probability. The target type I error probability was set at 0.05.

Table 7B gives the design operating characteristics under selected values of the true probability P_t. It gives the probability of stopping the trial under the alternative hypothesis, the expected sample size, and the average sample size given that the trial stopped. For example, if the true value of P_t is 0.7, then there is an 81% chance that the trial is stopped early and the average sample size is about 11.

Table 7A. Rate of toxicities: Testing rate $P_t > 0.4$.

Number of Patients	Number to Continue	Probability to Stop	Cumulative Probability to Stop
1	1	0	0
2	2	0	0
3	3	0	0
4	3	0.0256	0.0256
5	4	0	0.0256
6	5	0	0.0256
7	5	0.00983	0.03543
8	6	0	0.03543
9	7	0	0.03543
10	7	0.00425	0.03968
11	8	0	0.03968
12	9	0	0.03968
13	9	0.00199	0.04167
14	10	0	0.04167
15	10	0.00247	0.04414
16	11	0	0.04414
17	11	0.0026	0.04674
18	12	0	0.04674
19	12	0.00257	0.04932
20	13	0	0.04932
21	14	0	0.04932
22	14	0.00099	0.0503
23	15	0	0.0503

24	15	0.00117	0.05147
25	16	0	0.05147

Table 7B.

True Value of P_t	Probability to Stop	Expected N	Expected N given that we Stopped
0.1	0.0001	25	4.11
0.2	0.0018	24.96	4.51
0.3	0.0115	24.78	5.69
0.4	0.0515	24.13	8.15
0.5	0.1843	22.34	10.59
0.6	0.4726	18.65	11.57
0.7	0.8092	13.46	10.74

8.5 Statistical Analyses

Distributions of time-to-event data will be estimated with the nonparametric Kaplan-Meier estimator. Median progression-free survival at 6 and 12 months, time to progression, duration of response, and overall survival will be estimated from these distributions.

Although a phase II trial is unlikely to definitively establish whether a marker can be used to predict clinical benefit, we may identify an association that can then be further explored in definitive phase III trials [34, 35]. Therefore, the following analyses are of exploratory nature. Depending on the distribution of the expression levels of the selected biomarkers (PD-1, PD-L1, CD8, CD4, FoxP3 and Ki67), Pearson's and Spearman's rank correlation coefficients will be used to assess the pairwise association between them. Univariate analysis based on log-rank test will be used to evaluate the association between biomarkers measured as categorical variables and time-to-event outcomes. Univariate Cox proportional hazards regression models will be used to assess which biomarkers measured as continuous variables are predictive of patient time-to-event outcomes.

9.0 LABELING, PACKAGING, STORAGE AND RETURN OF COMMERCIAL SUPPLIES

9.1 Investigational Product

The investigator shall take responsibility for and shall take all steps to maintain appropriate records and ensure appropriate supply, storage, handling, distribution and usage of investigational product in accordance with the protocol and any applicable laws and regulations.

Commercial Supplies will be provided by Merck as summarized in Table 8.

Table 8 Product Descriptions

Product Name & Potency	Dosage Form
Pembrolizumab 50 mg	Lyophilized Powder for Injection
Pembrolizumab 100 mg/ 4mL	Solution for Injection

9.2 Packaging and Labeling Information

Commercial supplies will be affixed with a clinical label in accordance with regulatory requirements.

9.3 Commercial Supplies Disclosure

This trial is open-label; therefore, the subject, the trial site personnel, Merck, the Investigator and/or designee are not blinded to treatment. Drug identity (name, strength) is included in the label text; random code/disclosure envelopes or lists are not provided.

9.4 Storage and Handling Requirements

Commercial supplies must be stored in a secure, limited-access location under the storage conditions specified on the label.

Receipt and dispensing of trial medication must be recorded by an authorized person at the trial site.

Commercial supplies may not be used for any purpose other than that stated in the protocol.

9.5 Returns and Reconciliation

The investigator is responsible for keeping accurate records of the commercial supplies received from Merck or designee, the amount dispensed to and returned by the subjects and the amount remaining at the conclusion of the trial.

Upon completion or termination of the study, all unused and/or partially used investigational product will be destroyed at the site per institutional policy. It is the Investigator's responsibility to arrange for disposal of all empty containers, provided that procedures for proper disposal have been established according to applicable federal, state, local and institutional guidelines and procedures, and provided that appropriate records of disposal are kept.

10.0 STUDY MANAGEMENT

10.1 Conflict of Interest

Any reportable conflict of interest will be disclosed to the local IRB and will be outlined in the Informed Consent Form.

10.2 Institutional Review Board and Consent

It is expected that the IRB will have the proper representation and function in accordance with federally mandated regulations. The IRB should approve the consent form and protocol.

In obtaining and documenting informed consent, the investigator should comply with the applicable regulatory requirement(s), and should adhere to Good Clinical Practice (GCP) and to ethical principles that have their origin in the Declaration of Helsinki.

Before recruitment and enrollment onto this study, the patient will be given a full explanation of the study and will be given the opportunity to review the consent form. Each consent form must include all the relevant elements currently required by the FDA Regulations and local or state regulations. Once this essential information has been provided to the patient and the investigator is assured that the patient understands the implications of participating in the study, the patient will be asked to give consent to participate in the study by signing an IRB-approved consent form.

Prior to a patient's participation in the trial, the written informed consent form should be signed and personally dated by the patient and by the person who conducted the informed consent discussion.

10.3 Registration Procedures

All subjects that sign informed consent will be assigned a subject number sequentially by their date of consent. Those subjects that do not pass the screening phase will be listed as screen failures on the master list of consented subjects. Eligible subjects, as determined by screening procedures and verified by a treating investigator, will be registered on study at Cedars Sinai Medical Center by the Study Coordinator.

Issues that would cause treatment delays after registration should be discussed with the Principal Investigator (PI). If a patient does not receive protocol therapy following registration, the patient's registration on the study may be canceled. The Study Coordinator should be notified of cancellations as soon as possible.

The study team will track all subjects who sign consent on a subject screening/enrollment log using a unique screening ID (S001, S002, etc.). Subjects found to be ineligible will be recorded as screen failures. Subjects found to be eligible will be registered. Please see section 7.1.1.6 for more information on subject ID assignment.

A) <u>Eligibility Verification</u>

Prior to registration, all subjects must undergo eligibility verification by the SOCCI Clinical Research Office (CRO). The following documents will be completed and provided for review:

- Registration form (or equivalent)
- Copy of required laboratory tests
- Copy of required imaging reports
- Eligibility checklist (signed by investigator)
- Signed patient consent form and Subject's Bill of Rights
- HIPAA authorization form

B) <u>Registration</u>

After eligibility is verified, registration is completed as follows:

- Assign a patient study number
- Enter the patient in OnCore
- Notify the investigational pharmacy and treating physicians that a subject has gone on study and anticipated treatment start date

Oversight by the principal investigator is required throughout the entire registration process

11.0 DATA AND SAFETY MONITORING

11.1 Data Monitoring and Quality Assurance

Adherence to the protocol, Good Clinical Practices (GCP), and institutional policy will be monitored by the PI during the course of the study through routine Disease Research Group (DRG) meetings or equivalent. In addition, the SOCCI CRO Quality Management Core (QMC) will conduct the following:

- Audit preparations (AP) prior to audit conducted by any external auditing agency (i.e. NCI or FDA). The purpose an AP is to ensure adequate source documentation to support protocol compliance and data integrity are present and organized and to identify and correct any major findings prior to the external audit
- A thorough review of selected subject cases, regulatory files, and IP accountability records (if applicable) within 2-3 months after the first subject is enrolled and annually thereafter while subjects are receiving investigational intervention.
- Central eligibility verification for all subjects enrolled as described in protocol section 10.3.
- Central review of all eligibility waiver requests by a SOCCI Medical Reviewer to assess appropriateness and risk to ensure quality data and ensure subject safety protections for investigator-initiated research

For any protocol, QMC has the authority to request more frequent reviews or closer safety monitoring if it is deemed appropriate for any reason.

11.2 Safety Monitoring

Oversight of the progress and safety of the study will be provided by the PI. The PI will maintain continuous safety monitoring for the duration of the study by reviewing subject/study data. Adverse events and unanticipated problems are not expected, but if they occur they will be documented and reported according to CSMC IRB policies and procedures. If the PI becomes aware of any new safety information that may place subjects at increased risk than what was previously known the IRB will be promptly notified and if warranted, enrollment may be held until the PI determines whether a modification to the study is necessary and/or the informed consent documents are updated accordingly.

In addition, this protocol will utilize oversight by a Safety Committee On Early Phase Studies (SCOEPS). Committee membership includes experts in the field of oncology and early phase studies and biostatistics. SCOEPS' responsibilities are governed by the committee charter or equivalent.

The SCOEPS will provide routine monitoring of safety and enrollment for all early phase investigator-initiated trials (IITs). The committee meets routinely and is responsible for reviewing and adjudicating all dose-limiting toxicities, dose escalations and appropriateness of the escalation, cohort expansion, subject replacements, select AEs, SAEs, and confirmation of attainment of maximum tolerated dose.

The SCOEPS findings and recommendations will be reported in writing to the Principal Investigator. A summary report will be forwarded by the Principal Investigator or his/her designee to the Cedars-Sinai Medical Center IRB.

11.3 Adherence to the Protocol

Except for an emergency situation in which proper care for the protection, safety, and well-being of the study patient requires alternative treatment, or a protocol exception request approved by the SOCCI Medical Director and CSMC IRB, the study shall be conducted exactly as described in the approved protocol.

11.3.1 Emergency Modifications

Investigators may implement a deviation from, or a change of, the protocol to eliminate an immediate hazard(s) to trial subjects without prior IRB approval. For any such emergency modification implemented, the IRB must be notified as soon as possible, but no more than 10 days from the investigator's awareness of the event.

11.3.2 Protocol Exceptions and Eligibility Waivers

An exception is a type of planned deviation to the protocol, such as an eligibility waiver to enroll a subject who does not meet the approved inclusion/exclusion criteria. Unlike an amendment, a protocol exception most often involves a single subject and is not a permanent revision to the research protocol. The PI or her/his designee is responsible for submitting a protocol exception to the SOCCI Medical Director for review of the request and its supporting documentation to make a determination for further action. If the determination is an approval, then the request must be submitted in Webridge to the CSMC IRB. If the request is acknowledged by the IRB, then the protocol exception/eligibility waiver may be implemented.

Logistical deviations from the protocol (e.g., minor changes to the study schedule for an individual subject) do not require prior IRB approval unless the deviation has the potential to affect the subject's safety. Such planned deviations that do not affect the subject's safety should be noted in the subject's research record. Planned exceptions to the protocol that are more than logistical and/or have the potential to affect the subject's safety or study integrity may not be implemented without prior approval from the SOCCI Medical Director and IRB.

11.3.3 Other Protocol Deviations/Violations

Unintentional deviations from the protocol that might affect subject safety or study integrity should be reported to the IRB within 10 days from when the investigator becomes aware that such a deviation has occurred. In this case, a Protocol Deviation report must be submitted in Webridge. All submissions should include a description of the plan to avoid similar deviations in the future.

11.4 Amendments to the Protocol

Should amendments to the protocol be required, the amendments will be originated and documented by the Principal Investigator. It should also be noted that when an amendment to the protocol substantially alters the study design or the potential risk to the patient, a revised consent form might be required.

The written amendment, and if required the amended consent form, must be sent to the IRB for approval prior to implementation.

Repeat exceptions or deviations to the protocol may suggest a protocol amendment is needed.

11.5 Obligations of Investigators

The Principal Investigator is responsible for the conduct of the clinical trial at the site in accordance with Title 21 of the Code of Federal Regulations and/or the Declaration of Helsinki. The Principal Investigator is responsible for personally overseeing the treatment of all study patients. The Principal Investigator must assure that all study site personnel, including sub-investigators and other study staff members, adhere to the study protocol and all FDA/GCP/NCI regulations and guidelines regarding clinical trials both during and after study completion.

The Principal Investigator at each institution or site will be responsible for assuring that all the required data will be collected and entered onto the Case Report Forms and into the HIPAA-compliant study database. Periodically, monitoring visits will be conducted and the Principal Investigator will provide access to his/her original records to permit verification of proper entry of data. At the completion of the study, all case report forms will be reviewed by the Principal Investigator and will require his/her final signature to verify the accuracy of the data.

11.6 Record Retention

Study documentation includes all Case Report Forms, data correction forms or queries, source documents, monitoring logs/letters, and regulatory documents (e.g., protocol and amendments, IRB correspondence and approval, signed patient consent forms).

Source documents include all recordings of observations or notations of clinical activities and all reports and records necessary for the evaluation and reconstruction of the clinical research study.

Government agency regulations and directives require that the study investigator must retain all study documentation pertaining to the conduct of a clinical trial. Study documents should be kept on file until three years after the completion and final study report of this investigational study or as required by institutional guidelines.

12.0 REFERENCES

1. Zhang L, Conejo-Garcia JR, Katsaros D, Gimotty PA, Massobrio M, Regnani G, Makrigiannakis A, Gray H, Schlienger K, Liebman MN *et al*: **Intratumoral T cells, recurrence, and survival in epithelial ovarian cancer**. *N Engl J Med* 2003, **348**(3):203-213.
2. Ohtani H: **Focus on TILs: prognostic significance of tumor infiltrating lymphocytes in human colorectal cancer**. *Cancer immunity* 2007, **7**:4.
3. Ibrahim EM, Al-Foheidi ME, Al-Mansour MM, Kazkaz GA: **The prognostic value of tumor-infiltrating lymphocytes in triple-negative breast cancer: a meta-analysis**. *Breast Cancer Res Treat* 2014, **148**(3):467-476.
4. Gooden MJ, de Bock GH, Leffers N, Daemen T, Nijman HW: **The prognostic influence of tumour-infiltrating lymphocytes in cancer: a systematic review with meta-analysis**. *Br J Cancer* 2011, **105**(1):93-103.
5. Chen DS, Mellman I: **Oncology meets immunology: the cancer-immunity cycle**. *Immunity* 2013, **39**(1):1-10.
6. McGuire WP, Hoskins WJ, Brady MF, Kucera PR, Partridge EE, Look KY, Clarke-Pearson DL, Davidson M: **Cyclophosphamide and cisplatin compared with paclitaxel and cisplatin in patients with stage III and stage IV ovarian cancer**. *N Engl J Med* 1996, **334**(1):1-6.
7. Homet Moreno B, Ribas A: **Anti-programmed cell death protein-1/ligand-1 therapy in different cancers**. *Br J Cancer* 2015, **112**(9):1421-1427.
8. Brahmer JR, Tykodi SS, Chow LQ, Hwu WJ, Topalian SL, Hwu P, Drake CG, Camacho LH, Kauh J, Odunsi K *et al*: **Safety and activity of anti-PD-L1 antibody in patients with advanced cancer**. *N Engl J Med* 2012, **366**(26):2455-2465.
9. Hamanishi J, Mandai M, Ikeda T, Minami M, Kawaguchi A, Matsumura N, Abiko K, Baba T, Yamaguchi K, Ueda A *et al*: **Efficacy and safety of anti-PD-1 antibody (Nivolumab: BMS-936558, ONO-4538) in patients with platinum-resistant ovarian cancer.** In. J Clin Oncol 32:5s, 2014 (suppl; abstr 5511); 2014.
10. Hamanishi J, Mandai M, Ikeda T, Minami M, Kawaguchi A, Murayama T, Kanai M, Mori Y, Matsumoto S, Chikuma S *et al*: **Safety and Antitumor Activity of Anti-PD-1 Antibody, Nivolumab, in Patients With Platinum-Resistant Ovarian Cancer**. *J Clin Oncol* 2015.
11. Peters GJ, Bergman AM, Ruiz van Haperen VW, Veerman G, Kuiper CM, Braakhuis BJ: **Interaction between cisplatin and gemcitabine in vitro and in vivo**. *Semin Oncol* 1995, **22**(4 Suppl 11):72-79.
12. Rose PG, Mossbruger K, Fusco N, Smrekar M, Eaton S, Rodriguez M: **Gemcitabine reverses cisplatin resistance: demonstration of activity in platinum- and multidrug-resistant ovarian and peritoneal carcinoma**. *Gynecol Oncol* 2003, **88**(1):17-21.
13. Nagourney RA, Brewer CA, Radecki S, Kidder WA, Sommers BL, Evans SS, Minor DR, DiSaia PJ: **Phase II trial of gemcitabine plus cisplatin repeating doublet therapy in previously treated, relapsed ovarian cancer patients**. *Gynecol Oncol* 2003, **88**(1):35-39.

14. Brewer CA, Blessing JA, Nagourney RA, Morgan M, Hanjani P: **Cisplatin plus gemcitabine in platinum-refractory ovarian or primary peritoneal cancer: a phase II study of the Gynecologic Oncology Group**. *Gynecol Oncol* 2006, **103**(2):446-450.
15. Emens LA: **Chemoimmunotherapy**. *Cancer journal* 2010, **16**(4):295-303.
16. van der Most RG, Robinson BW, Lake RA: **Combining immunotherapy with chemotherapy to treat cancer**. *Discovery medicine* 2005, **5**(27):265-270.
17. Rosenberg SA, Yang JC, Restifo NP: **Cancer immunotherapy: moving beyond current vaccines**. *Nat Med* 2004, **10**(9):909-915.
18. Cho BK, Rao VP, Ge Q, Eisen HN, Chen J: **Homeostasis-stimulated proliferation drives naive T cells to differentiate directly into memory T cells**. *J Exp Med* 2000, **192**(4):549-556.
19. Goldrath AW, Bogatzki LY, Bevan MJ: **Naive T cells transiently acquire a memory-like phenotype during homeostasis-driven proliferation**. *J Exp Med* 2000, **192**(4):557-564.
20. Drake CG: **Combination immunotherapy approaches**. *Ann Oncol* 2012, **23 Suppl 8**:viii41-46.
21. Tseng CW, Hung CF, Alvarez RD, Trimble C, Huh WK, Kim D, Chuang CM, Lin CT, Tsai YC, He L *et al*: **Pretreatment with cisplatin enhances E7-specific CD8+ T-Cell-mediated antitumor immunity induced by DNA vaccination**. *Clin Cancer Res* 2008, **14**(10):3185-3192.
22. Ramakrishnan R, Assudani D, Nagaraj S, Hunter T, Cho HI, Antonia S, Altiok S, Celis E, Gabrilovich DI: **Chemotherapy enhances tumor cell susceptibility to CTL-mediated killing during cancer immunotherapy in mice**. *J Clin Invest* 2010, **120**(4):1111-1124.
23. Suzuki E, Kapoor V, Jassar AS, Kaiser LR, Albelda SM: **Gemcitabine selectively eliminates splenic Gr-1+/CD11b+ myeloid suppressor cells in tumor-bearing animals and enhances antitumor immune activity**. *Clin Cancer Res* 2005, **11**(18):6713-6721.
24. Duffy AG, Greten TF: **Immunological off-target effects of standard treatments in gastrointestinal cancers**. *Ann Oncol* 2014, **25**(1):24-32.
25. Antonia SJ, Brahmer JR, Gettinger SN, Quan Man Chow L, Juergens RA, Shepherd FA, Laurie SA, Gerber DE, Goldman JW, Shen Y *et al*: **Nivolumab (anti-PD-1; BMS-936558, ONO-4538) in combination with platinum-based doublet chemotherapy (PT-DC) in advanced non-small cell lung cancer (NSCLC).** In. J Clin Oncol 32:5s, 2014 (suppl; abstr 8113); 2014.
26. Weber JS, Yang JC, Atkins MB, Disis ML: **Toxicities of Immunotherapy for the Practitioner**. *J Clin Oncol* 2015, **33**(18):2092-2099.
27. Lynch TJ, Bondarenko I, Luft A, Serwatowski P, Barlesi F, Chacko R, Sebastian M, Neal J, Lu H, Cuillerot JM *et al*: **Ipilimumab in combination with paclitaxel and carboplatin as first-line treatment in stage IIIB/IV non-small-cell lung cancer: results from a randomized, double-blind, multicenter phase II study**. *J Clin Oncol* 2012, **30**(17):2046-2054.
28. Seymour, L, Bogaerts J, Perrone A, Ford R, *et al*: iRECIST: guidelines for response criteria for use in trials testing immunotherapeutics. Lancet Oncol 2017 Mar;18(3):e143-e152.

29. Aaronson NK, Ahmedzai S, Bergman B, Bullinger M, Cull A, Duez NJ, Filiberti A, Flechtner H, Fleishman SB, de Haes JC *et al*: **The European Organization for Research and Treatment of Cancer QLQ-C30: a quality-of-life instrument for use in international clinical trials in oncology**. *J Natl Cancer Inst* 1993, **85**(5):365-376.
30. Smith TJ, Bohlke K, Lyman GH, Carson KR, Crawford J, Cross SJ, Goldberg JM, Khatcheressian JL, Leighl NB, Perkins CL *et al*: **Recommendations for the Use of WBC Growth Factors: American Society of Clinical Oncology Clinical Practice Guideline Update**. *J Clin Oncol* 2015, **33**(28):3199-3212.
31. Simon R: **Optimal two-stage designs for phase II clinical trials**. *Control Clin Trials* 1989, **10**(1):1-10.
32. Pazdur R: **Endpoints for assessing drug activity in clinical trials**. *Oncologist* 2008, **13 Suppl 2**:19-21.
33. Eisenhauer EA, Therasse P, Bogaerts J, Schwartz LH, Sargent D, Ford R, Dancey J, Arbuck S, Gwyther S, Mooney M *et al*: **New response evaluation criteria in solid tumours: revised RECIST guideline (version 1.1)**. *Eur J Cancer* 2009, **45**(2):228-247.
34. McShane LM, Hunsberger S, Adjei AA: **Effective incorporation of biomarkers into phase II trials**. *Clin Cancer Res* 2009, **15**(6):1898-1905.
35. Dancey JE, Dobbin KK, Groshen S, Jessup JM, Hruszkewycz AH, Koehler M, Parchment R, Ratain MJ, Shankar LK, Stadler WM *et al*: **Guidelines for the development and incorporation of biomarker studies in early clinical trials of novel agents**. *Clin Cancer Res* 2010, **16**(6):1745-1755.

APPENDICES

APPENDIX A: ECOG PERFORMANCE STATUS

Grade	Description
0	Normal activity. Fully active, able to carry on all pre-disease performance without restriction.
1	Symptoms, but ambulatory. Restricted in physically strenuous activity, but ambulatory and able to carry out work of a light or sedentary nature (e.g., light housework, office work).
2	In bed <50% of the time. Ambulatory and capable of all self-care, but unable to carry out any work activities. Up and about more than 50% of waking hours.
3	In bed >50% of the time. Capable of only limited self-care, confined to bed or chair more than 50% of waking hours.
4	100% bedridden. Completely disabled. Cannot carry on any self-care. Totally confined to bed or chair.
5	Dead.
* As published in Am. J. Clin. Oncol.: *Oken, M.M., Creech, R.H., Tormey, D.C., Horton, J., Davis, T.E., McFadden, E.T., Carbone, P.P.: Toxicity And Response Criteria Of The Eastern Cooperative Oncology Group. Am J Clin Oncol 5:649-655, 1982.* The Eastern Cooperative Oncology Group, Robert Comis M.D., Group Chair.	

APPENDIX B: CTCAE

Common Terminology Criteria for Adverse Events V4.0 (CTCAE)

The descriptions and grading scales found in the revised NCI Common Terminology Criteria for Adverse Events (CTCAE) version 4.0 will be utilized for adverse event reporting. (http://ctep.cancer.gov/reporting/ctc.html)

APPENDIX C: RECIST 1.1

Response Evaluation Criteria in Solid Tumors (RECIST) Version 1.1

C1. Measurability of tumor at baseline

Measurable disease.

Tumor lesions: must be accurately measured in at least one dimension (longest diameter in the plane of measurement is to be recorded) with a minimum size of:

- 10 mm by CT scan (CT scan slice thickness no greater than 5 mm)
- 10 mm caliper measurement by clinical exam (lesions which cannot be accurately measured with calipers should be recorded as non-measurable)
- 20 mm by chest x-ray

Malignant lymph nodes: to be considered pathologically enlarged and measurable, a lymph node must be ≥ 15 mm in short axis when assessed by CT scan (CT scan slice thickness recommended to be no greater than 5 mm). At baseline and in follow-up, only the short axis will be measured and followed.

Non-measurable disease. All other lesions, including small lesions (longest diameter < 10 mm or pathological lymph nodes with ≥ 10 to < 15 mm short axis) as well as truly non-measurable lesions). Lesions considered truly non-measurable include: leptomeningeal disease, ascites, pleural/pericardial effusions, inflammatory breast disease, lymphangitic involvement of skin or lung, abdominal masses/abdominal organomegaly identified by physical exam that is not measurable by reproducible imaging techniques.

C2. Methods of Measurements

All measurements should be recorded in metric notation, using calipers if clinically assessed. All baseline evaluations should be performed as closely as possible to the beginning of treatment and never more than 28 days before the beginning of the treatment.

The same method of assessment and the same technique should be used to characterize each identified and reported lesion at baseline and during follow-up. Imaging-based evaluation should always be done rather than clinical examination unless the lesion(s) being followed cannot be imaged but are assessable by clinical exam.

C3. Baseline Documentation of 'target' and 'non-target' lesions

Target lesions. All measurable lesions up to a maximum of 2 lesions per organ and 5 lesions in total, representative of all involved organs, should be identified as **target lesions** and recorded and measured at baseline and at the specified interval during treatment. Target lesions should be selected on the basis of their size (lesions with the longest diameter), be representative of all involved organs, but in addition should be those that lend themselves to reproducible repeated measurements. A sum of the diameters (longest for non-nodal lesions, short axis for nodal lesions) for all target lesions will be calculated and reported as the baseline sum diameters. The baseline sum diameters will be used as reference by which to characterize the objective tumor response.

Non-target lesions. All other lesions (or sites of disease) should be identified as **non-target lesions** and should also be recorded at baseline. Measurements of these lesions are not required and these lesions should be followed as 'present', 'absent' or in rare cases 'unequivocal progression'.

C4. Tumor Response Evaluation

Evaluation of Target Lesions

Complete Response (CR): Disappearance of all target lesions. Any pathological lymph nodes (whether target or non-target) must have reduction in short axis to < 10 mm.

Partial Response (PR): At least a 30% decrease in the sum of the diameters of target lesions, taking as reference the baseline sum diameters.

Progressive Disease (PD): At least a 20% increase in the sum of the diameters of target lesions, taking as reference the smallest sum on study (this includes the baseline sum if that is the smallest sum on study). In addition to the relative increase of 20%, the sum must also demonstrate an absolute increase of at least 5 mm. Note: the appearance of one or more new lesions is also considered progression.

Stable Disease (SD): Neither sufficient shrinkage to qualify for PR nor sufficient increase to qualify for PD, taking as reference the smallest sum of diameters while on study.

Special note on assessment of lymph nodes: When lymph nodes are included as target lesion, the 'sum' of lesions may not be zero even if complete response criteria are met, since a normal lymph node is defined as having a short axis < 10 mm. In order to qualify ofr CR, each node must achieve a short axis of < 10 mm. For PR, SD and PD, the actual short axis measurement of the nodes is to be included in the sum of target lesions.

Target lesions that become 'too small to measure': If the radiologist believes the lesion has likely disappeared, the measurement should be recorded as 0 mm. If a lesion is believed to be present but is too small to measure, a default value of 5 mm should be assigned. If the radiologist is able to provide an actual measure, that should be recorded, even if it is below 5 mm.

Lesions that split or coalesce on treatment: When non-nodal lesions 'fragment', the longest diameters of the fragmented portions should be added together to calculate the target lesion sum. As lesions coalesce, a plane between them may be maintained that would aid in obtaining maximal diameter measurement of each individual lesion. If the lesions have truly coalesced such that the are no longer separable, the vector of the longest diameter in this instance should be the maximal longest diameter for the 'coalesced lesion'.

Evaluation of Non-Target Lesions

Complete Response (CR): Disappearance of all non-target lesions and normalization of tumor marker level. All lymph nodes must be non-pathological in size (<10 mm short axis).

Non-CR/Non-PD: Persistence of one or more non-target lesions and/or maintenance of tumor marker level above the normal limits,

Progressive Disease (PD): unequivocal progression of existing non-target lesions or the appearance of one or more lesions.

The cytological confirmation of the neoplastic origin of any effusion that appears or worsens during treatment when the measurable tumor has met criteria for response or stable disease is mandatory to differentiate between response or stable disease or progressive disease.

C5. Evaluation of Response

When both target and non-target lesions are present, individual assessments will be recorded separately. Determination of tumor response at each assessment is summarized in the following table:

Target Lesions	Non-target Lesions	New Lesions	Overall Response
CR	CR	No	CR
CR	Non-CR/Non-PD	No	PR
CR	Not evaluated	No	PR
PR	Non-PD or not all evaluated	No	PR
SD	Non-PD or not all evaluated	No	SD
Not all evaluated	Non-PD	No	NE
PD	Any	Yes or No	PD
Any response	PD	Yes or No	PD
Any response	Any response	Yes	PD

The best overall response is the best response recorded from the start of the treatment until the end of treatment taking into account any requirement for confirmation. To be assigned a status of PR or CR, changes in tumor measurements with responding tumors should be confirmed by repeat imaging performed at least 4 weeks after the criteria for response are first met. In the case of SD, follow-up measurement must have met the SD criteria at least once after study entry at a minimum interval of 6 weeks.

RECIST version 1.1* will be used in this study for assessment of tumor response. While either CT or MRI may be utilized, as per RECIST 1.1, CT is the preferred imaging technique in this study.

* As published in the European Journal of Cancer [33]

APPENDIX D: IMMUNE-RELATED RESPONE CRITERIA

iRECIST Guidelines [28]

Immunotherapeutic agents can sometimes cause an initial increase in tumor burden or the appearance of new lesions. The iRECIST criteria were developed as an adaptation to RECIST criteria to account for this observation, iRECIST will be assessed secondarily following the addition of pembrolizumab to treatment. iRECIST utilizes the following adaptations to RECIST 1.1.

	RECIST 1.1	iRECIST
Definitions of measurable and non-measurable disease; numbers and site of target disease	Measurable lesions are ≥10 mm in diameter (≥15 mm for nodal lesions); maximum of five lesions (two per organ); all other disease is considered non-target (must be ≥10 mm in short axis for nodal disease)	No change from RECIST 1.1; however, new lesions are assessed as per RECIST 1.1 but are recorded separately on the case report form (but not included in the sum of lesions for target lesions identified at baseline)
Complete response, partial response, or stable disease	Cannot have met criteria for progression before complete response, partial response, or stable disease	Can have had iUPD (one or more instances), but not iCPD, before iCR, iPR, or iSD
Confirmation of complete response or partial response	Only required for non-randomised trials	As per RECIST 1.1
Confirmation of stable disease	Not required	As per RECIST 1.1
New lesions	Result in progression; recorded but not measured	Results in iUPD but iCPD is only assigned on the basis of this category if at next assessment additional new lesions appear or an increase in size of new lesions is seen (≥5 mm for sum of new lesion target or any increase in new lesion non-target); the appearance of new lesions when none have previously been recorded, can also confirm iCPD
Independent blinded review and central collection of scans	Recommended in some circumstances—eg, in some trials with progression-based endpoints planned for marketing approval	Collection of scans (but not independent review) recommended for all trials
Confirmation of progression	Not required (unless equivocal)	Required
Consideration of clinical status	Not included in assessment	Clinical stability is considered when deciding whether treatment is continued after iUPD

"i" indicates immune responses assigned using iRECIST. RECIST=Response Evaluation Criteria in Solid Tumours. iUPD=unconfirmed progression. iCPD=confirmed progression. iCR=complete response. iPR=partial response. iSD=stable disease.

***Table 1:* Comparison of RECIST 1.1 and iRECIST**

APPENDIX E: FACIT-TS-G QUESTIONNAIRE

FACIT-TS-G (Version 1)

Please evaluate your experience on this treatment: ______________________________
(name of treatment)

If you have not completed your treatment, please answer the questions the best you can. All of your answers will be kept confidential.

Please mark one answer for each of the following questions.

		A lot worse	A little worse	About the same	A little better	A lot better
TS1	Compared to what you expected, how do you rate the <u>effectiveness of the treatment</u> so far?	0	1	2	3	4
TS2	Compared to what you expected, how do you rate the <u>side effects of treatment</u> so far?	0	1	2	3	4

		No, not at all	Yes, to some extent	Yes, for the most part	Yes, completely
TS3	Did your doctor(s) help you evaluate the effects of your treatment so far?......................................	0	1	2	3
TS4	Do you feel you received the treatment that was right for you?	0	1	2	3
TS5	Are you satisfied with the effects of this treatment so far?	0	1	2	3

		No	Maybe	Yes
TS6	Would you recommend this treatment to others with your illness?..	0	1	2
TS7	Would you choose this treatment again?..............	0	1	2

		Poor	Fair	Good	Very Good	Excellent
TS8	How do you rate this treatment overall?	0	1	2	3	4

Thank you! Do you have any comments? ______________________________
____

APPENDIX F: FACT-G QUESTIONNAIRE

FACT-G (Version 4)

Below is a list of statements that other people with your illness have said are important. **Please circle or mark one number per line to indicate your response as it applies to the past 7 days.**

	PHYSICAL WELL-BEING	Not at all	A little bit	Some-what	Quite a bit	Very much
GP1	I have a lack of energy	0	1	2	3	4
GP2	I have nausea	0	1	2	3	4
GP3	Because of my physical condition, I have trouble meeting the needs of my family	0	1	2	3	4
GP4	I have pain	0	1	2	3	4
GP5	I am bothered by side effects of treatment	0	1	2	3	4
GP6	I feel ill	0	1	2	3	4
GP7	I am forced to spend time in bed	0	1	2	3	4

	SOCIAL/FAMILY WELL-BEING	Not at all	A little bit	Some-what	Quite a bit	Very much
GS1	I feel close to my friends	0	1	2	3	4
GS2	I get emotional support from my family	0	1	2	3	4
GS3	I get support from my friends	0	1	2	3	4
GS4	My family has accepted my illness	0	1	2	3	4
GS5	I am satisfied with family communication about my illness	0	1	2	3	4
GS6	I feel close to my partner (or the person who is my main support)	0	1	2	3	4
Q1	*Regardless of your current level of sexual activity, please answer the following question. If you prefer not to answer it, please mark this box ☐ and go to the next section.*					
GS7	I am satisfied with my sex life	0	1	2	3	4

FACT-G (Version 4)

Please circle or mark one number per line to indicate your response as it applies to the past 7 days.

	**EMOTIONAL WELL-BEING**	**Not at all**	**A little bit**	**Some-what**	**Quite a bit**	**Very much**
GE1	I feel sad	0	1	2	3	4
GE2	I am satisfied with how I am coping with my illness	0	1	2	3	4
GE3	I am losing hope in the fight against my illness	0	1	2	3	4
GE4	I feel nervous	0	1	2	3	4
GE5	I worry about dying	0	1	2	3	4
GE6	I worry that my condition will get worse	0	1	2	3	4

	**FUNCTIONAL WELL-BEING**	**Not at all**	**A little bit**	**Some-what**	**Quite a bit**	**Very much**
GF1	I am able to work (include work at home)	0	1	2	3	4
GF2	My work (include work at home) is fulfilling	0	1	2	3	4
GF3	I am able to enjoy life	0	1	2	3	4
GF4	I have accepted my illness	0	1	2	3	4
GF5	I am sleeping well	0	1	2	3	4
GF6	I am enjoying the things I usually do for fun	0	1	2	3	4
GF7	I am content with the quality of my life right now	0	1	2	3	4

APPENDIX G: Dose Modification and Toxicity Management Guidelines for Immune-related AEs Associated with Pembrolizumab

General instructions:

1. Corticosteroid taper should be initiated upon AE improving to Grade 1 or less and continue to taper over at least 4 weeks.
2. For situations where pembrolizumab has been withheld, pembrolizumab can be resumed after AE has been reduced to Grade 1 or 0 and corticosteroid has been tapered. Pembrolizumab should be permanently discontinued if AE does not resolve within 12 weeks of last dose or corticosteroids cannot be reduced to ≤10 mg prednisone or equivalent per day within 12 weeks.
3. For severe and life-threatening irAEs, IV corticosteroid should be initiated first followed by oral steroid. Other immunosuppressive treatment should be initiated if irAEs cannot be controlled by corticosteroids.

Immune-related AEs	Toxicity grade or conditions (CTCAEv4.0)	Action taken to pembrolizumab	irAE management with corticosteroid and/or other therapies	Monitor and follow-up
Pneumonitis	Grade 2	Withhold	• Administer corticosteroids (initial dose of 1-2 mg/kg prednisone or equivalent) followed by taper	• Monitor participants for signs and symptoms of pneumonitis<br>• Evaluate participants with suspected pneumonitis with radiographic imaging and initiate corticosteroid treatment<br>• Add prophylactic antibiotics for opportunistic infections
	Grade 3 or 4, or recurrent Grade 2	Permanently discontinue		
Diarrhea / Colitis	Grade 2 or 3	Withhold	• Administer corticosteroids (initial dose of 1-2 mg/kg prednisone or equivalent) followed by taper	• Monitor participants for signs and symptoms of enterocolitis (ie, diarrhea, abdominal pain, blood or mucus in stool with or without fever) and of bowel perforation (ie, peritoneal signs and ileus).<br>• Participants with ≥ Grade 2 diarrhea suspecting colitis should consider GI consultation and performing endoscopy to rule out colitis.<br>• Participants with diarrhea/colitis should be advised to drink liberal quantities of clear fluids. If sufficient oral fluid intake is not feasible, fluid and electrolytes should be substituted via IV infusion.
	Grade 4	Permanently discontinue		

AST / ALT elevation or Increased bilirubin	Grade 2	Withhold	• Administer corticosteroids (initial dose of 0.5- 1 mg/kg prednisone or equivalent) followed by taper	• Monitor with liver function tests (consider weekly or more frequently until liver enzyme value returned to baseline or is stable
	Grade 3 or 4	Permanently discontinue	• Administer corticosteroids (initial dose of 1-2 mg/kg prednisone or equivalent) followed by taper	
Type 1 diabetes mellitus (T1DM) or Hyperglycemia	Newly onset T1DM or Grade 3 or 4 hyperglycemia associated with evidence of β-cell failure	Withhold	• Initiate insulin replacement therapy for participants with T1DM<br>• Administer anti-hyperglycemic in participants with hyperglycemia	• Monitor participants for hyperglycemia or other signs and symptoms of diabetes.
Hypophysitis	Grade 2	Withhold	• Administer corticosteroids and initiate hormonal replacements as clinically indicated.	• Monitor for signs and symptoms of hypophysitis (including hypopituitarism and adrenal insufficiency)
	Grade 3 or 4	Withhold or permanently discontinue[1]		
Hyperthyroidism	Grade 2	Continue	• Treat with non-selective beta-blockers (eg, propranolol) or thionamides as appropriate	• Monitor for signs and symptoms of thyroid disorders.
	Grade 3 or 4	Withhold or permanently discontinue[1]		
Hypothyroidism	Grade 2-4	Continue	• Initiate thyroid replacement hormones (eg, levothyroxine or liothyroinine) per standard of care	• Monitor for signs and symptoms of thyroid disorders.
Nephritis and Renal dysfunction	Grade 2	Withhold	• Administer corticosteroids (prednisone 1-2 mg/kg or equivalent) followed by taper.	• Monitor changes of renal function
	Grade 3 or 4	Permanently discontinue		
Myocarditis	Grade 1 or 2	Withhold	• Based on severity of AE administer corticosteroids	• Ensure adequate evaluation to confirm etiology and/or exclude other causes
	Grade 3 or 4	Permanently discontinue		

All other immune-related AEs	Intolerable/ persistent Grade 2	Withhold	• Based on type and severity of AE administer corticosteroids	• Ensure adequate evaluation to confirm etiology and/or exclude other causes
	Grade 3	Withhold or discontinue based on the type of event. Events that require discontinuation include and not limited to: Gullain-Barre Syndrome, encephalitis		
	Grade 4 or recurrent Grade 3	Permanently discontinue		

1. Withhold or permanently discontinue pembrolizumab is at the discretion of the investigator or treating physician.

NOTE:

For participants with Grade 3 or 4 immune-related endocrinopathy where withhold of pembrolizumab is required, pembrolizumab may be resumed when AE resolves to ≤ Grade 2 and is controlled with hormonal replacement therapy or achieved metabolic control (in case of T1DM).

APPENDIX H: Summary of Protocol Changes

IIT2015-13-Walsh-PemCiGem Summary of Changes

Amending Version 1 dated 8DEC2015 received IRB approval on February 4, 2016.

Amendment 1: The following changes were made primarily to accommodate the use of new Quality of Life questionnaires (FACIT-TS-G/FACT-G) and delete reference to EORTC QLQ.

1. Title page
   A. Version date updated
2. Table of Contents – Updated to include new Quality of Life questionnaires (FACIT-TS-G/FACT-G) to replace EORTC QLQ
3. List of Abbreviations – Updated to include new Quality of Life questionnaires (FACIT-TS-G/FACT-G) to replace EORTC QLQ
4. Section 2.1 – Updated to include new Quality of Life questionnaires (FACIT-TS-G/FACT-G) to replace EORTC QLQ
5. Section 4.2.3.3 – Updated to include new Quality of Life questionnaires (FACIT-TS-G/FACT-G) to replace EORTC QLQ
6. Section 6.1 – Updated to include new Quality of Life questionnaires (FACIT-TS-G/FACT-G) to replace EORTC QLQ
7. Section 7.0
   A. Section 7.1.2.7 – Added line that shipping instructions will come from Qualtek, the outside lab processing the tissue samples.
   B. Section 7.1.3.1 – Deleted reference to Qualtek, as they will not be processing the blood samples.
8. Section 12 – Updated to include new Quality of Life questionnaires (FACIT-TS-G/FACT-G) reference
9. Appendices – Added Appendix E and F, new Quality of Life questionnaires (FACIT-TS-G/FACT-G) to replace EORTC QLQ (formerly Appendix E)

Amending Protocol Version 2 dated 05FEB2016 received IRB approval on February 11, 2016.

Amendment 2: The following changes were made primarily to accommodate the use of pre-medications sooner than previously written. Other protocol clarifications and edits are included in the list below.

1. Title page – Version date updated
2. List of Abbreviations – CSF (colony-stimulating factor) added
3. Section 4.2.3.2 – Updated to clarify biomarker endpoints.
4. Section 5.1.1.3 – Added “platinum-based” for internal consistency in defining platinum-resistant ovarian cancer.
5. Section 5.1.1.5 – Added ECOG performance status of 2 as eligible as this is more inclusive of individuals with platinum-resistant ovarian cancer.
6. Section 5.2.1.2 – The text regarding parameters for chemotherapy dose adjustments was re-formatted into a new table, Table 3C: Guidelines for chemotherapy dose modifications for increased clarity. Toxicities resulting in dose modifications were specifically defined to also increase clarity. For example, “grade 4 thrombocytopenia” was replaced with “platelet count < 25,000/microliter”.

7. Section 5.2.1.2 – A section was added to allow for growth factor support as recommended by the 2015 American Society of Clinical Oncology Clinical Practice Guideline. The prior language that restricted growth factor support use to after a second episode of febrile neutropenia was removed as this was too restrictive, posed a safety concern, and did not reflect current guidelines for standard of care growth factor support use. A protocol exception was approved by Dr. Sandler and the IRB on 3/10/2016 to allow for growth factor support to a patient who experienced febrile neutropenia during cycle 1 of chemotherapy in this study.
8. Section 5.2.3 – the language surrounding antiemetic and hydration regimens was modified to be less restrictive and more reflective of current standard of care. A statement was added to allow for the addition of decadron 10 mg IV as an antiemetic premedication. This was approved by Merck on 2/29/2016. A protocol exception was approved by the IRB on 3/1/2016 to allow a subject to receive decadron as an antiemetic premedication during cycle 2 of chemotherapy in this study.
9. Section 5.5.2 – this language was updated to allow for use of decadron 10 mg IV as an antiemetic premedication.
10. Section 6.0 – days were removed from the first row of the study flow chart as these were potentially inaccurate given the administrative leeway allowing for +/-3 days on day 1 and +/-1 day on day 8 of therapy. "+21" was added to cycles 2-6 and maintenance to reflect the fact that these are 21 day cycles.
11. Section 7.1.1.6 – clarification was provided on the assignment of patient numbers.
12. Section 7.1.1.9 – a return carriage was added to indicate that this is a new section.
13. Section 7.1.2 – the language was updated to clarify the timing of assessments. Exam, vital signs, weight, assessment of adverse events and medication review may be performed during a clinic visit in between cycles. Laboratory studies can be performed up to 72 hours prior to treatment (this is internally consistent with existing language on page 43 of the protocol).
14. Section 7.1.2.3 – the language was updated to clarify the allowed timing of directed physical exam.
15. Section 7.1.2.6 – the language was updated to clarify alternative imaging assessment for patients with allergy to IV contrast.
16. Section 10.3 – add a line referencing section 7.1.1.6 for more information on subject registration.
17. Section 11.1 – the Data and Quality Assurance language was updated to reflect current SOCCI practice.
18. Section 11.2 – subsection title was updated to delete the word data. This section only addresses current SOCCI practice for safety review of IITs, the Safety Committee On Early Phase Studies.
19. Section 11.2.1 – the original section was duplicative of the text in subsection 11.1 and is now deleted.
20. Section 11.2.2 – this section was deleted as it is no longer relevant. IMM was replaced by SCOEPS.
21. Section 11.3 – a line was added to clarify that an approved protocol exception is an instance when the protocol is not adhered to in addition to an emergency modification.
22. Section 11.3.1 – some text was deleted and is now included in the following sections 11.3.2 and 11.3.3.
23. Section 11.3.2 – new section was added to clarify when an exception or an eligibility waiver may be implemented.
24. Section 11.3.3 – formerly section 11.3.2 was edited to only address protocol deviations.

25. Section 11.4 – a line was added to clarify that a protocol amendment may be necessary if there are repeated exceptions or deviations.
26. Section 11.5 – added a line to include data entry into a HIPAA-compliant database is also a study obligation of the PI.

Amending Protocol Version 3 dated 18MAR2016 received IRB approval on March 30, 2016.

Amendment 3: Changes were made to correct discrepancies within the protocol that required reporting serious events within 24 hours to the IRB. CSMC IRB policy requires reporting of unanticipated or serious events to the IRB occur within 10 days of discovering the event. Other protocol clarifications and edits are included in the list below.

1. Title page

   Version number and date updated

   Independent Medical Monitor deleted – safety monitoring section was updated in Version 3

2. Table of Contents: added Appendix G Summary of Changes

3. Section 2.1: clarified that maintenance cycles are for up to an additional 11 cycles, totaling approximately 1 year for study treatment, not an additional 1 year of maintenance cycles

4. Section 4.2.3.2: clarified that biopsy and blood collections are optional, but donation of archival tissue is not if research biopsy is not performed

5. Section 5.1

   Subsection 5.1.1.5: deleted ECOG 2, subjects with an ECOG status of 0 or 1 will be enrolled

   Subsection 5.1.1.9: added inclusion criterion that subjects must donate archival tissue if they opt out of a biopsy for a fresh sample

6. Section 5.2.1.2: added a treatment parameter for grade 3 or 4 toxicity to Dose Modification Table 3C

7. Section 5.7.3: reference to “reporting within 24 hours to the IRB” deleted

8. Section 6.0: Clarifications made to cycle header, questionnaire time points, and magnesium was combined with other SOC blood draws that occur on Day 1 and Day 8 of treatment

   Footnote clarification added: the discontinuation visit and all follow-up are only necessary if a subject was administered pembrolizumab

   Footnote clarification added: Medication review, Adverse Event review, and the Physical Exam, can take place up to 7 days prior to treatment

   Footnote clarification added: Only FACT-G questionnaire will be administered at the baseline/screening visit. The FACIT-TS-G is not applicable prior to treatment

Footnote added: CBC with diff, CMPL and magnesium are done on day 1 and day 8

9. Section 7.1.3: PK was deleted from Table 5

10. Section 7.1.4.1: added description of questionnaires

11. Section 7.1.5:

    7.1.5.2: clarified that the maintenance phase is up to an additional 11 cycles, totaling approximately one year of study treatment

    7.1.5.3.1 and 7.1.5.4 sentence added to two subsections to clarify that follow-up visits are only required if subjects received pembrolizumab

12. Section 7.2: all references to "reporting within 24 hours to the IRB" were deleted

13. Section 7.3.1: clarified that *study staff* must report an SAE to the PI

14. Section 7.3.3: deleted section that required reporting SAEs to the FDA. This study is IND exempt.

15. Table 6: clarified that an overdose should be reported to Merck within 2 business days

16. Appendices: appended Summary of Changes – Appendix G

Amending Protocol Version 4 dated 13MAY2016 received IRB approval on May 25, 2016.

Amendment 4: See changes below.

1. Title page: Version number and date updated

2. Eligibility Criteria:
   Section 5.1.1.3 – histologically confirmed deleted, as stated in Administrative Memo #3
   Section 5.1.2.9 – criterion regarding pneumonitis was updated to match sponsor's current safety language. See Dear Investigator Letter for Pembrolizumab MISP - Action Requested date July 25, 2016 submitted with this amendment.

3. Section 7.2.6: Added line that the study will only collect lab abnormalities if they are graded a 3 or 4, as stated in Administrative Memo #4.

Protocol Version 6, dated 16FEB2017, amends Protocol Version 5. IRB approval on 15 March 2017

Amendment 5: See changes below:

1. Exclusion criteria expands exclusion criteria 5.1.2.5 to include the note: Patients who have hypertension as an adverse event related to prior angiogenesis targeted therapy may be allowed if ≤ Grade 2 and considered by investigator to be well-controlled on anti-hypertensive agents.

2. "Clinical Supplies" changed to "Commercial Supplies" throughout the protocol.

Protocol Version 7, dated 11July2017, amends Protocol Version 6. IRB approval on 01OCT2017

Amendment 6: See changes below:

1. Changes to CT Scan window to allow for out of window CT Scans based on clinical indication.
2. Clarification changes to section 6.0 (study events table)
3. Clarifications made throughout document

Protocol Version 8, dated 10OCT2017, amends Protocol Version 7. IRB approval on 21NOV2017

Amendment 7: See changes below:

1. **Section 4.2.3.2**
   - Edits to language throughout this section to clarify biomarker research.
   - 6-week intervals clarified to every two cycles

2. **Section 4.2.3.3**
   - Edits to language to clarify QOL questionnaire time points.

3. **Section 5.1.1.7**
   - Clarification that patients who have had prior hysterectomy and/or bilateral oophorectomy are not required to have a pregnancy test.

4. **Section 5.2.1 (Table 3C)**
   - Changes to dose modification to specify that only the drug(s) related to specific drug toxicity will be held instead of entire treatment:

     - Continued administration of pembrolizumab/hold of cisplatin and gemcitabine chemo if *"DAY 1: ANC < 1500 cells/mm3 and/or platelet count < 100,000/µl"* treatment to be modified as follows: *"Cycle 1 and 2: Hold cisplatin and gemcitabine chemotherapy. Initiation of cycle 1 or 2 of chemotherapy will be delayed a maximum of three weeks until these threshold values are achieved. Subjects who fail to recover adequate counts within a three-week delay will not receive further chemotherapy in the study."* Protocol previously indicated hold of pembrolizumab in addition to chemotherapy.

     - Clarification of continued administration of gemcitabine and/or pembrolizumab, hold cisplatin if scheduled if *"Grade 2 or greater peripheral neuropathy (moderate symptoms, limiting instrumental activities of daily living)"* or *"Grade 2 or greater renal toxicity (serum creatinine greater than 1.8 mg/dL or 1.5X baseline)"*

  - Continued administration of cisplatin and/or pembrolizumab if scheduled, delayed gemcitabine if subject experiences Grade 3 or greater liver toxicity, until toxicity resolved to Grade 1.

  - Continued administration of pembrolizumab/hold of cisplatin and gemcitabine chemo if *"Grade 3 or 4 toxicity attributable to chemotherapy."* Protocol previously indicated hold of pembrolizumab in addition to chemotherapy.

5. **Section 5.2.3**
   - Addition of +/- 10 minutes to chemotherapy administration window.

6. **Section 6.1 (Study flowchart)**
   - Addition of CT imaging SOC time points to study flowchart to clarify CT window.
   - Clarification that labs on day 8 are only required if patient is scheduled to receive cisplatin and gemcitabine treatment.
   - Clarification that archival tissue will not be required during screening phase.
   - Clarification that patients who have had prior hysterectomy and/or bilateral oophorectomy are not required to have a pregnancy test.
   - Clarification that Laboratory studies may be performed up to 72 hours prior to treatment (Section 7.1.2). Labs do not need to be repeated if day 1 treatment is delayed or held unless clinically indicated per PI.

7. **Section 7.1.2.7**
   - Clarification that Collection of archival tissue is not required during the screening phase

8. **Section 7.1.3**

   - Clarification to laboratory testing review: *"If review is found to be acceptable, the investigator or qualified designee will enter an order to release treatment (no other specific documentation will be required)."*

9. **Section (Appendix C)**
   - Update of RESIST to match Version 1.1

Protocol Version 9, dated 30NOV2017, amends Protocol Version 8. IRB approval on XX XXX XXXX

Amendment 8: See changes below:

- Addition of Appendix G: Dose Modification and Toxicity Management Guidelines for Immune-related AEs Associated with Pembrolizumab

----

Protocol Version 10, dated 01FEB2018, amends Protocol Version 9

Amendment 9: See changes below:

1. Drug administration changed from 1 year to 2 year period (6 cycles of combined treatment + 28 cycles of maintenance)

2. Section 4.2.3.3 Quality of Life Questionnaires
   - (FACT-G and FACIT-TS-G) additional time point at 24 months (after cycle 34)

3. Section 6.1 Study Flow Chart
   - Revised Maintenance cycles to include up to Cycle 34
   - Imaging revised to every third cycle starting with Cycle 9
   - QOL questionnaires additional time point at Cycle 34
   - Pembro administration extended through Cycle 34

Protocol Version 11, dated 02Jan2019, amends Protocol Version 10

Amendment 10: See changes below:

1. Removal of Co-Investigator: Beth Karlan, MD
   - Title page updated

2. Protocol updated to include additional the allowance for decadron at 10 or 20 mg IV doses per Merck Approval
   - Section 5.2.3 Timing of Dose Administration
   - Section 5.5.2 Prohibited Concomitant Medications

3. Protocol clarified to indicate iRECIST will be used rather than irRC or irRECIST
   - Page 14 – “irRC” changed to “iRECIST”
   - Page 23 – “irRECIST” changed to “iRECIST”
   - Page 61 – correct reference added
   - Page 68 – Appendix corrected to reflect iRECIST criteria

4. Subject replacement strategy updated
   - Page 32 – “will” changed to “may”

5. Protocol clarified for consistency regarding grade 1 and 2 laboratory abnormalities
   - Page 43 – “with the exception of grade 1 or 2 laboratory abnormalities” added
   - Page 46 – typo grade “3” corrected to “2”
